# Photo-generated dinuclear {Eu(II)}_2_ active sites for selective CO_2_ reduction in a photosensitizing metal-organic framework

**DOI:** 10.1038/s41467-018-05659-7

**Published:** 2018-08-22

**Authors:** Zhi-Hao Yan, Ming-Hao Du, Junxue Liu, Shengye Jin, Cheng Wang, Gui-Lin Zhuang, Xiang-Jian Kong, La-Sheng Long, Lan-Sun Zheng

**Affiliations:** 1 Collaborative Innovation Center of Chemistry for Energy Materials, State Key Laboratory of Physical Chemistry of Solid Surface and Department of Chemistry, College of Chemistry and Chemical Engineering, Xiamen University, 361005 Xiamen, China; 2 State Key Laboratory of Molecular Reaction Dynamics and Collaborative Innovation Center of Chemistry for Energy Materials, Dalian Institute of Chemical Physics, Chinese Academy of Sciences, 116023 Dalian, China; 3 College of Chemical Engineering, Zhejiang University of Technology, 310032 Hangzhou, China

## Abstract

Photocatalytic reduction of CO_2_ is a promising approach to achieve solar-to-chemical energy conversion. However, traditional catalysts usually suffer from low efficiency, poor stability, and selectivity. Here we demonstrate that a large porous and stable metal-organic framework featuring dinuclear Eu(III)_2_ clusters as connecting nodes and Ru(phen)_3_-derived ligands as linkers is constructed to catalyze visible-light-driven CO_2_ reduction. Photo-excitation of the metalloligands initiates electron injection into the nodes to generate dinuclear {Eu(II)}_2_ active sites, which can selectively reduce CO_2_ to formate in a two-electron process with a remarkable rate of 321.9 μmol h^−1^ mmol_MOF_^−1^. The electron transfer from Ru metalloligands to Eu(III)_2_ catalytic centers are studied via transient absorption and theoretical calculations, shedding light on the photocatalytic mechanism. This work highlights opportunities in photo-generation of highly active lanthanide clusters stabilized in MOFs, which not only enables efficient photocatalysis but also facilitates mechanistic investigation of photo-driven charge separation processes.

## Abstract (web summary)

Solar-to-chemical CO_2_ reduction provides a means to use light’s energy for CO_2_ removal and upgrading to useful products, although this photochemical conversion is challenging. Here, authors construct a Europium-containing metal-organic framework that selectively converts CO_2_ to formate with light.

## Introduction

The ever-increasing atmospheric carbon dioxide (CO_2_) level due to fossil fuel consumption raises growing concerns about global warming^1,2^. Therefore, developing new technology for CO_2_ capture and conversion is receiving considerable research interest. In this context, photocatalytic reduction of CO_2_ into renewable fuels is a promising strategy for solar-to-chemical energy conversion by using artificial photosynthetic systems^3–5^. This approach not only uses CO_2_ as C_1_ feedstock but also allows harvesting energy from sunlight, helping the transition towards a more sustainable energy source. During the last few decades, diverse inorganic semiconductors have been developed for carbon fixation, such as TiO_2_^6^, CdS^7^, ZnO^8^, and ZnGa_2_O_4_^9^, which were synthesized as photocatalysts to reduce CO_2_. However, the efficiencies of these materials are limited by their large band gaps, low densities of active sites on surfaces, and fast recombination rates of photo-generated electron–hole pairs^10–13^. Therefore, exploring new photocatalysts with enhanced efficiency for solar-driven CO_2_ reduction is highly desirable.

Metal-organic frameworks (MOFs), one type of crystalline porous hybrid materials, have attracted widespread attention due to their tailorable chemistry, uniform but tunable porosity, and high surface areas^14–18^. Up to now, great efforts have been dedicated to the synthesis and catalytic applications of porous MOFs. Recently, several MOFs have been taken as heterogeneous catalysts for photocatalytic reduction of CO_2_, including NH_2_-MIL-125(Ti)^19^, NH_2_-UiO-66(Zr)^20^, and porphyrin-MOFs^3,21^, some of which adopt the connecting metal clusters as the active sites. These metal clusters play a significant role on the photocatalytic activity of catalysts in photoreduction of CO_2_. Studies on lanthanide chemistry have shown that Eu(II) ion is highly active in reductive conversions^22,23^. We envisioned that introducing Eu(III) clusters as metal connecting nodes in MOFs followed up with photo-activation can generate isolated Eu(II) active cluster sites for CO_2_ reduction. On the other hand, ruthenium-polypyridine compounds are often used as photosensitizer for photocatalytic CO_2_ reduction due to their tremendous oxidation and reduction power and extended lifetimes of their excited states^24–26^. Studies by García et al.^27^ and Majima et al.^28^ revealed that the organic ligands in MOFs can serve as antenna for the metal clusters. Photoexcited electron transfer from ligands to catalytic centers is also observed. These previous studies lead us to hypothesize that integrating Ru-polypyridine photosensitizers into Eu cluster-based MOFs will be a promising strategy to enhance the catalytic activities on CO_2_ reduction under visible-light irradiation.

Here, we design an Eu-Ru(phen)_3_-MOF (phen = phenanthroline) by integrating the triangular Ru(phen)_3_-derived tricarboxylate ligand as photosensitizer into Eu-MOF with Eu_2_(μ_2_-H_2_O) secondary building units (SBUs). Interestingly, the Eu-Ru(phen)_3_-MOF exhibits visible-light-driven selective CO_2_ photoreduction to formate with a remarkable rate of 321.9 μmol h^−1^ mmol_MOF_^−1^. Noteworthily, such a self-assembled Eu-Ru(phen)_3_-MOF is the solitary example that exhibits a high efficiency for selective CO_2_ photo-reduction in the family of Ln-MOFs. Time-resolved photoluminescence (PL) spectroscopy combined with femtosecond transient optical absorption spectroscopy confirms that charge transfers from Ru photocenters to Eu-O cluster on a time scale of 1 to 300 ns. Moreover, in situ electron paramagnetic resonance (EPR) study clearly indicates that after accepting of photoexcited electrons from metalloligand, the Eu(III)_2_ clusters become active catalytic centers for the photoreduction of CO_2_.

## Results

### Synthesis and structural determination of Eu-Ru(phen)_3_-MOF

**Fig. 1:**
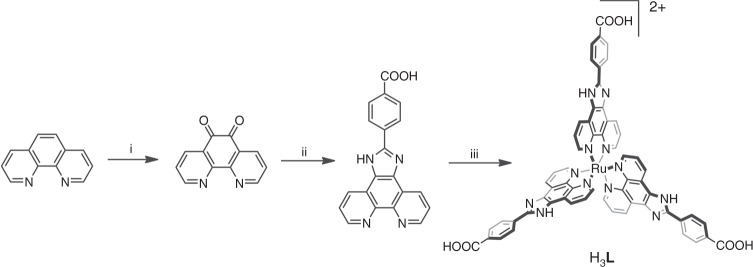
Synthesis of H_3_**L**. Chemical structure of the tricarboxylate metalloligand used in the synthesis of Eu-Ru(phen)_3_-MOF. (i) HNO_3_, H_2_SO_4_, KBr, NaOH, 90 °C, 96% yield; (ii) 4-carboxybenzaldehyde, HAc, 100 °C, NH_4_Ac, 120 °C, 88% yield; (iii) RuCl_3_·3H_2_O, EG, 180 °C, KFP_6_(aq), NaOH(aq), THF, EtOH, 80 °C, 87% yield

**Fig. 2:**
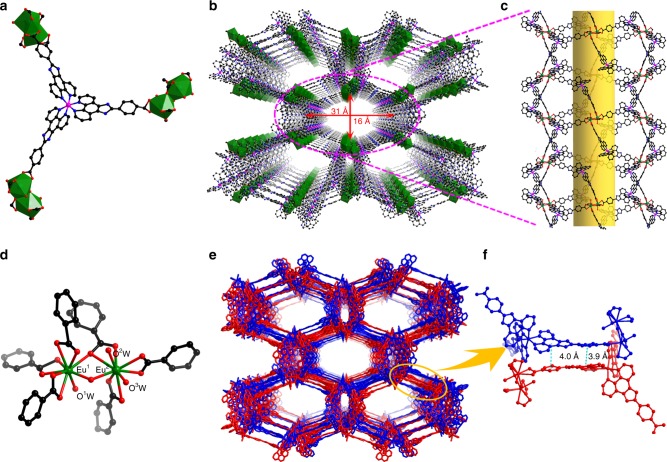
X-ray crystal structure of Eu-Ru(phen)_3_-MOF. **a** Stick/polyhedra model structure of the metalloligand. **b** Stick model representation of a single 3D framework viewed along the [010] direction showing the 1D channels **c** with window dimensions of 31 × 16 Å. **d** Ball-and-stick model of [Eu_2_(μ_2_-H_2_O)(H_2_O)_3_(-COO^−^)_6_] building unit in Eu-Ru(phen)_3_-MOF. **e** Stick model showing the interpenetrated frameworks in Eu-Ru(phen)_3_-MOF and **f** the two neighboring networks stabilized by the π–π stacking interactions

The triangular Ru(phen)_3_-derived tricarboxylate acid metalloligand (H_3_**L**) was prepared from 1,10-phenanthroline in a multistep sequence in a 73% overall yield, as shown in Fig. 1 (Supplementary Figs. 1–7 and Supplementary Methods). The resultant Eu-Ru(phen)_3_-MOF formulated as [Eu_2_(μ_2_-H_2_O)(H_2_O)_3_(L)_2_]·(NO_3_)_2_·(2-FBA^−^)_2_·(H_2_O)_22_ was synthesized by a reaction of Eu(NO_3_)_3_·6H_2_O, H_3_**L**, and 2-fluorobenzoate (2-FBA) in dimethylformamide (DMF) at 105 °C for 70 h (small light red block-shaped crystals in 44% yield). Single-crystal X-ray crystallographic study with synchrotron radiation reflected that the Eu-Ru(phen)_3_^−^MOF crystallize in a orthorhombic crystal system with space group of *I*_222_. The MOF adopts a structure of twofold interpenetrated coordination networks. Within each of the framework, the propeller-like metalloligands with *D*_3_ symmetry and six-connected (6-c) [Eu_2_(μ_2_-H_2_O)(H_2_O)_3_(-COO^−^)_6_] SBUs (Fig. 2a) linked to each other alternately to generate a three-dimensional (3D) framework containing one-dimensional 16 × 31 Å channels along the [010] direction (Fig. 2b, c). The three-connected (3-c) metalloligand and the 6-c {Eu(III)}_2_ cluster (Fig. 2d) are linked together, leading to a (3,6)-connected *scu*-type topology with the (4•6^2^)^2^(4^2^•6^9^•8^4^) point symbol (Supplementary Fig. 8). Two sets of the symmetric net interpenetrated into each other, resulting in a twofold interpenetrated structure (Fig. 2e). There are two types of interconnected channels in the Eu-Ru(phen)_3_-MOF structure: one is a continuous channel along the [011] direction, with window dimensions of 15 × 20 Å, and the other one with smaller aperture is along the [111] direction (Supplementary Fig. 9). As depicted in Fig. 2e, f, the π–π stacking interactions between metalloligands stabilized the adjacent interpenetrated frameworks. The void space in the MOF was calculated to be 74.5% by PLATON^29^. The amount of nitrogen gas adsorption of Eu-Ru(phen)_3_-MOF at 77 K is far less than that predicted from the structure (Supplementary Fig. 10) due to distortion of the framework during drying process (Supplementary Note 1)^30,31^. Based on thermogravimetric analysis (TGA) (Supplementary Fig. 11) and charge balance, there are two NO_3_^−^ and two 2-FBA anions in the channel of the Eu-Ru(phen)_3_-MOF.

### Photocatalytic CO_2_ reduction

**Fig. 3:**
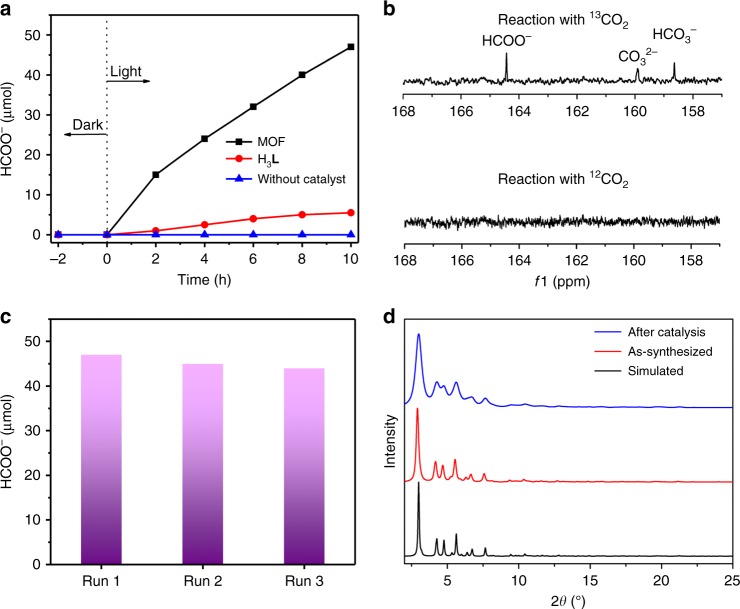
Photocatalytic CO_2_ reduction performance. **a** Time profiles of HCOO^−^ produced catalyzed by Eu-Ru(phen)_3_-MOF or H_3_**L** or without catalyst under irradiation with a Xe lamp (420–800 nm). **b** The ^13^C NMR spectrum of products in liquid phase after reacting with ^13^CO_2_ and ^12^CO_2_, respectively. **c** The amount of HCOO^−^ produced for reusing three times. Samples were recovered after each cycle and reused under identical reaction conditions. **d** PXRD patterns for as-synthesized Eu-Ru(phen)_3_-MOF and after photocatalytic reaction, showing its well-retained structure during the catalysis

The photocatalytic CO_2_ reduction activity was tested under visible-light irradiation (420 nm < *λ* < 800 nm) by using Eu-Ru(phen)_3_-MOF as heterogeneous photocatalyst and triethanolamine (TEOA) as sacrificial agent (Supplementary Fig. 12). The concentration of formate HCOO^−^ product in the liquid phase was quantified by using ion chromatograph. As shown in Fig. 3a, under continuous visible-light illumination, formate production exhibits a time-dependent increase. The amount of generated HCOO^−^ reached 47 μmol in 10 h with the average formation rate of HCOO^−^ of 321.9 μmol h^−1^ mmol_MOF_^−1^ (mmol_MOF_ calculated from its SBUs, Supplementary Table 1). This value is higher than those of previous catalyst bast on MOF materials, such as NH_2_-MIL-125(Ti), NH_2_-UiO-66(Zr), and PCN-222 under similar conditions (the formation rates of HCOO^−^ for these photocatalysts are 26.5, 46.3, and 143.5 μmol h^−1^ mmol_MOF_^−1^, respectively)^19–21^, and some visible-light responsive semiconductors^32,33^. The higher photocatalytic activity should be attributed to the introduction of a photosensitizing and efficient light-harvesting Ru(phen)_3_ moiety in this system. In addition, no CO or H_2_ or CH_4_ products in the gas or liquid phases was observed (Supplementary Figs. 13 and 14), suggesting that Eu-Ru(phen)_3_-MOF has high selectivity in reducing CO_2_ to formate. The control experiments showed that no HCOO^−^ was produced either without Eu-Ru(phen)_3_-MOF, TEOA, or in dark (Supplementary Fig. 15a). To evaluate the photocatalytic stability, recycling experiments of photocatalytic CO_2_ reduction in MeCN/TEOA (*v:v* = 20:1) were performed (Supplementary Fig. 15b). As shown in Fig. 3a, after 10 h illumination, the HCOO^−^ amount was about 47 μmol and no noticeable degradation after three consecutive reactions (Fig. 3c). Meanwhile, the PXRD patterns after photocatalytic reactions match well with those of as-prepared sample, suggesting the stability of Eu-Ru(phen)_3_-MOF after photocatalytic reaction (Fig. 3d). The scanning electron microscope (SEM) images show that morphology of the Eu-Ru(phen)_3_-MOF changed after photocatalytic reaction as a result of mechanical stirring (Supplementary Fig. 16). Moreover, inductively coupled plasma-mass spectrometry (ICP-MS) results indicates <0.1% metal leaching to the solution after photocatalytic reaction of 10 h, confirming the stability of Eu-Ru(phen)_3_-MOF in photocatalysis.

The control experiment using ^13^CO_2_ as reactant was studied to validate the source of HCOO^−^ product, and the generated H^13^COO^−^ was detected by ^13^C NMR spectroscopy. As shown in Fig. 3b, after reaction of 6 h, the ^13^C NMR spectrum clearly displays three peaks at 125.7, 159.8, and 164.4 ppm, corresponding to CO_2,_ HCO_3_^−^, and HCOO^−^ respectively. In contrast, these three peaks were absent in the ^13^C NMR spectrum when ^12^CO_2_ was used as the reactant (Supplementary Fig. 17), unambiguously demonstrating that Eu-Ru(phen)_3_-MOF indeed promotes the photocatalytic CO_2_ reduction. In the photocatalytic reaction, TEOA were oxidized to its aldehyde form (Supplementary Figs. 18 and 19, Supplementary Note 2).

### Absorption and PL analyses

**Fig. 4:**
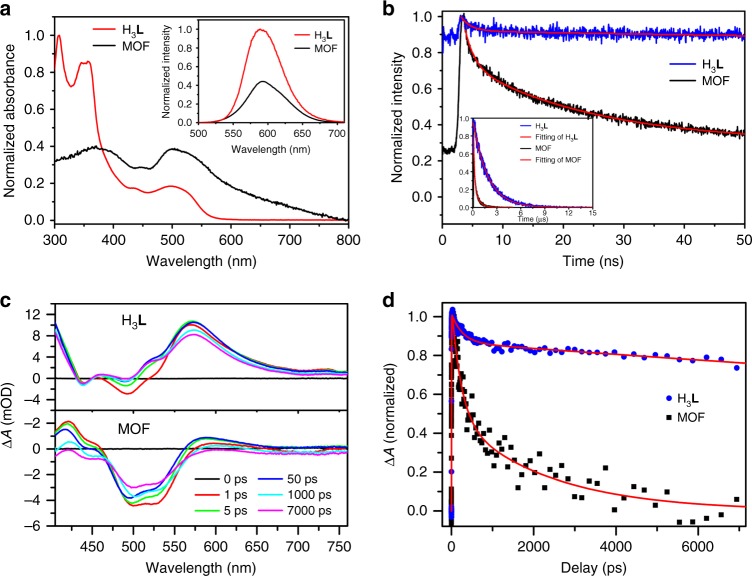
Spectroscopic evidence for effective electron transfer process. **a** Normalized UV–vis of Eu-Ru(phen)_3_-MOF and H_3_**L** in DMF. Inset: Emission spectra of Eu-Ru(phen)_3_-MOF and H_3_**L** (*λ*_ex_ = 465 nm). **b** Normalized luminescence decay traces of Eu-Ru(phen)_3_-MOF and H_3_**L** over the first 50 ns (*λ*_ex_ = 377 nm). Inset: Decay transients measured at 630 nm (*λ*_ex_ = 465 nm). **c** Transient absorption spectra of Eu-Ru(phen)_3_-MOF and H_3_**L** at various time delays, and **d** corresponding kinetic traces at 604 nm

To reveal the photoinduced charge transport mechanism, we investigated the samples by using Ultraviolet–visible (UV–vis) absorption, static, and time-resolved emission spectra. As shown in Fig. 4a, Eu-Ru(phen)_3_-MOF and H_3_**L** both showed strong absorption bands at 300–350 nm because of the π–π* transition of phen ligand. The relatively weaker absorption peaks in the region of 420–480 nm correspond to the characteristic broad absorption of Ru^2+^-centered metal-to-ligand charge transfer (MLCT) transitions. This similarity between H_3_**L** ligand and MOF suggested that the coordination of Eu^3+^ with H_3_**L** has no significant effect on the excitation energy of the latter^34,35^. However, the emission of H_3_**L** centered at ~590 nm, which represents the Ru(phen)_3_-centered triplet ^3^MLCT state, is significantly quenched after Eu_2_ coordination in MOF (Fig. 4a). Consistently, the time-resolved PL collected in the time window of <1 ns to 15 μs (Fig. 4b) of MOF shows much faster decay kinetics than H_3_**L**. These results suggest possible electron and/or energy transfer from H_3_**L** to Eu_2_ oxo-clusters^36^. Notably, no characteristic Eu^3+^ *f*–*f* emission were observed in Eu-Ru(phen)_3_-MOF, implying that the quenching and fast decay of Eu-Ru(phen)_3_-MOF emission should be due to electron transfer rather than energy transfer from H_3_**L** to Eu *f*–*f* transitions. To quantitatively estimate the electron transfer rate, the time-resolved PL kinetics are fitted by a bi-exponential function, as shown in Supplementary Tables 2 and 3. An electron transfer time ranging from 6.1 ns to 293.6 ns is determined (Supplementary Note 3).

### Ultrafast transient absorption and EPR characterizations

**Fig. 5:**
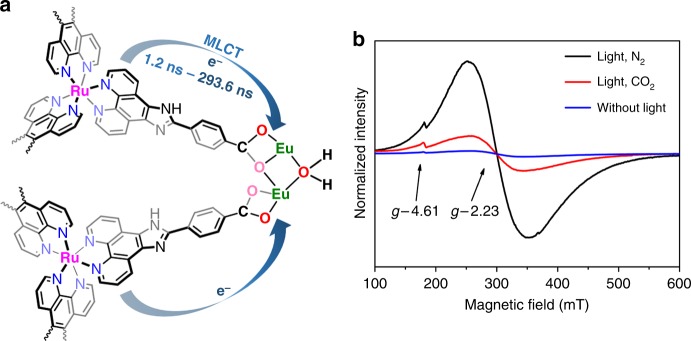
Photocatalytic in situ EPR characterization. **a** Schematic light-induced dynamics of Eu-Ru(phen)_3_-MOF based on the initial excitation of the Ru photocenter and the pathways of electron transfer from Ru to catalytic Eu_2_ oxo-cluster center. **b** In situ EPR spectra of Eu-Ru(phen)_3_-MOF under different conditions

To further confirm the photoinduced electron transfer kinetics in MOF, ultrafast transient absorption (TA) measurements were also carried out (the details of TA experiments in Supplementary Methods)^37–39^. In Fig. 4c, we show the comparison of the TA spectra between H_3_**L** ligand and Eu-Ru(phen)_3_-MOF at different delay times after 400 nm excitation. The TA spectra of H_3_**L** exhibits negative ground state bleach (GSB) signal at ~440 and ~490 nm, which overlaps with a strong and broad (440–700 nm) excited state absorption (ESA) signal (positive). In contrast, the ESA amplitude is significantly reduced in the MOF sample, leaving a more prominent and long-lived GSB signal. This spectroscopic feature further confirms the electron transfer from H_3_**L** to the coordinated Eu metal node rather than energy transfer, because in the latter case the GSB recovery and ESA decay should occur simultaneously. The TA kinetics of H_3_**L** and MOF probed at ESA (604 nm) are shown in Fig. 4d. The ESA signal in MOF shows a considerably faster decay, which reflects the electron transfer process. By fitting the kinetics by a bi-exponential function (Supplementary Table 4), we determined the electron transfer time of 1.2 ns. This transfer time is consistent with the faster time rate observed in time-resolved PL measured within 50 ns windows (Fig. 4b). The results of TA and time-resolved PL indicate that the electron transfer from H_3_**L** to the Eu_2_ oxo-clusters occurs on a broad time scale, ranging from a nanosecond to hundreds of nanosecond (Fig. 5a). However, according to the significant change of ESA signal in TA spectra, which decays by 90% in MOF vs. 20% in H_3_**L**, the electron transfer process should occur mainly within a few nanosecond time, which is consistent with reported electron transfer time in similar Ru–Pt complex^40^. Nevertheless, the observation of a wide range of electron transfer time suggest that the electron transfer process in Ru(phen)_3_-MOF may occurs along different transport pathways.

Following the electron transfer process for H_3_**L** ligand to the Eu metal, the recovery of GSB in the TA spectra of MOF sample reflects the back electron transfer from Eu metal node to H_3_**L** ligand, which brings the H_3_**L** cation to ground state. Within our TA time window of 7 ns, GSB signal recovers by only 30%, suggesting that the back electron transfer time is much longer than 7 ns. The slow back electron transfer thus ensures an effective charge separation in the MOF for CO_2_ reduction.

To obtain further insight into the photocatalytic process of CO_2_ reduction, in situ EPR experiments were studied as well, which can elucidate the photo-induced electron injection process. Under visible-light irradiation in a N_2_ atmosphere, the EPR spectrum of H_3_**L** shows a signal at *g* = 2.04. Meanwhile, when the light source was turned off, the signal quenching was observed immediately reflecting the visible-light-induced radical formation of the metalloligand and the subsequent charge transfer process (Supplementary Fig. 20). For Eu-Ru(phen)_3_-MOF, no EPR signal was observed without irradiation. Upon 2 min of visible-light irradiation, a broad EPR signal with *g* = 2.23 was observed, simultaneously a weak EPR signal was found at 4.61 (Fig. 5b). These two EPR signals are attributed to paramagnetic Eu^2+^ species, because the Eu^3+^ ions have no EPR signals, while the Eu^2+^ ions are EPR active^41,42^. The valence change of the Eu_2_ oxo-clusters can be attributed to the photo-induced LMCT process. Subsequently, when CO_2_ was introduced into the irradiated Eu-Ru(phen)_3_-MOF, the EPR signal corresponding to Eu^2+^ was greatly weakened due to some of the Eu^2+^ oxidized back to Eu^3+^ during the CO_2_ reduction process.

### DFT calculations

**Fig. 6:**
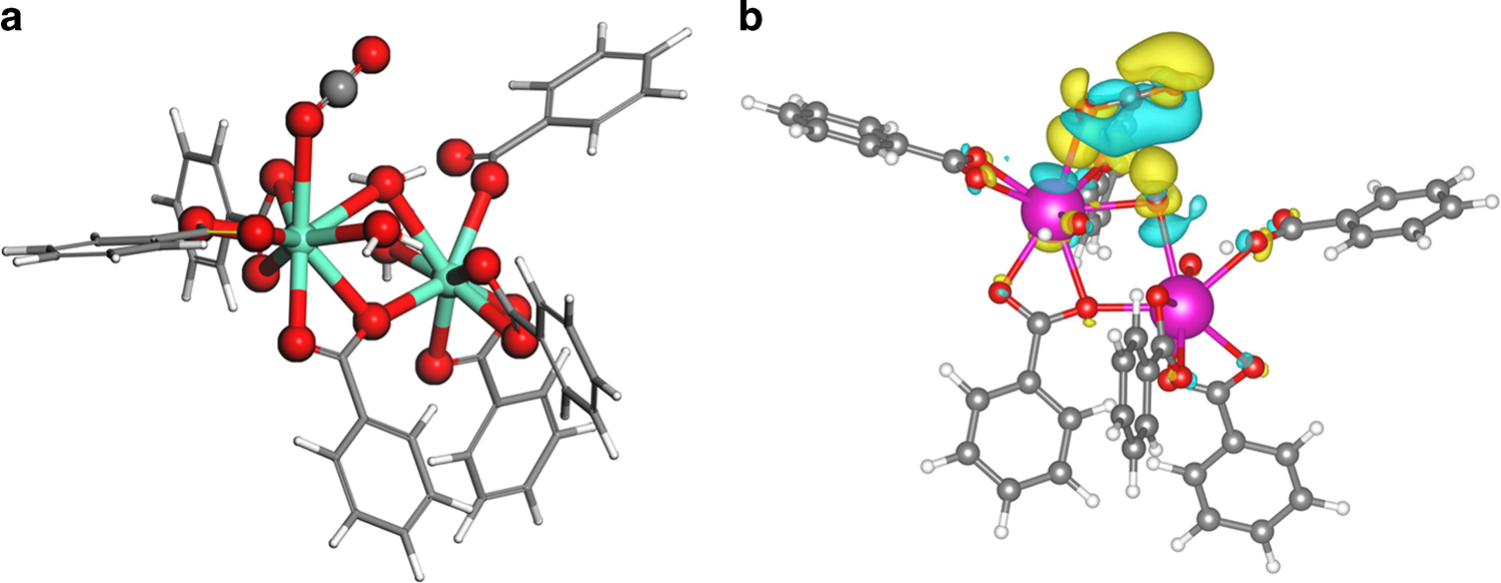
Density functional theory calculation. **a** The calculated CO_2_ adsorption structure. **b** Charge difference density of CO_2_ adsorption structure of Eu(II)_2_

To probe the active site of Eu-Ru(phen)_3_-MOF, the spin polarized density functional theory (DFT) calculations were studied using VASP program with Hubbard U correction (Supplementary Methods)^43,44^. In order to optimize the structure of Eu-Ru(phen)_3_-MOF, all hydrogen atoms were relaxed under the constraint of non-hydrogen atoms. The Eu_2_ unit derived from the structure was used as the computational model. The optimized structure reflects the Eu1-Ow1 bond that is weaker than the others, with an enthalpy difference of 0.80 eV. Therefore, removal of the Ow1 can generate a Lewis acid site of Eu(III), which is possibly facilitated by visible-light irradiation. The exposed Eu(III) center can adsorb one CO_2_ molecule through Eu-O linkage. Figure 6a illustrates the position of the CO_2_ adsorbed on the active site of Eu1 ion (*d*_(Eu1-O)_ = 2.745 Å), which was obtained from geometry optimization using DFT. The adsorption energy of CO_2_ was estimated to be −0.55 eV. After reducing the Eu(III) to Eu(II) via photo-induced electron injection, electron can be further transferred to CO_2_. The difference map of charge density reveals that CO_2_ molecule can obtain 0.025 electrons from the Eu(II)_2_ dimer (Fig. 6b)^45^, while Eu(II)1 and Eu(II)2 ions lose 0.0073 and 0.004 electrons, respectively, suggesting an effective activation of CO_2_ molecule.

## Discussion

**Fig. 7:**
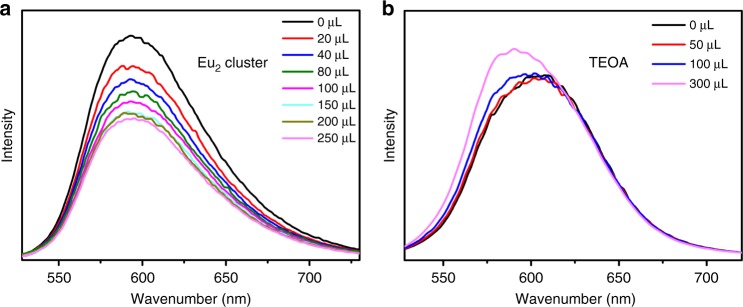
Fluorescence quenching. **a** Emission spectra of H_3_**L** after the addition of different amounts of [Eu_2_(MMA)_6_(H_2_O)_4_] and **b** TEOA in DMF with 465 nm excitation

To investigate the photocatalytic mechanism, photophysical and electrochemical studies were performed. To establish whether the excited [Ru^II^(phen)_3_]^2+^ was quenched reductive by TEOA or oxidative by Eu_2_ clusters, the luminescence quench experiments of H_3_**L** was studied with addition of the solution of TEOA or Eu_2_ clusters in DMF. The discrete Eu_2_ clusters with a similar structure to that of Eu_2_ SBUs in Eu-Ru(phen)_3_-MOF can be synthesized with a formula of [Eu_2_(MMA)_6_(H_2_O)_4_] (MAA = methacrylic acid)^46^ (Supplementary Fig. 21 and Supplementary Methods). As shown in Fig. 7a, the luminescence of H_3_**L** was quenched by the Eu_2_ moieties efficiently but not by TEOA (Fig. 7b). These results indicated that the photocatalytic reduction of CO_2_ process occurred via electron transfer from the photoexcited [Ru^II^(phen)_3_]^2+^ to Eu_2_ SBUs, but not from TEOA to the excited [Ru^II^(phen)_3_]^2+^. In the oxidative quenching, the generated [Ru^III^(phen)_3_]^3+^ was reduced by TEOA subsequently.

**Fig. 8:**
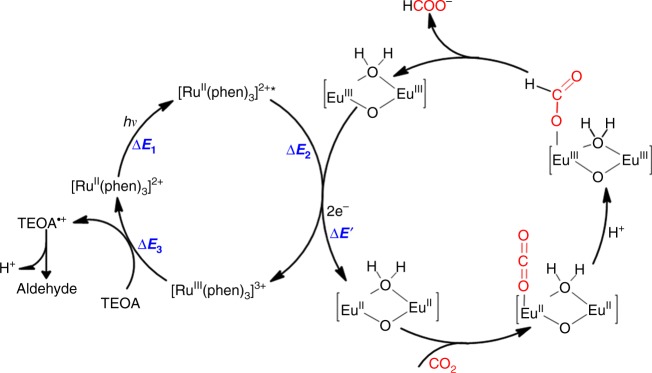
Proposed catalytic mechanism of photocatalytic CO_2_ reduction to HCOOH. The photo-initiated electron transfers from Ru photocenters to dinuclear Eu_2_ oxo-clusters in Eu-Ru(phen)_3_-MOF lead to the photo-reduction of CO_2_. Δ*E*_1_ = 2.07 eV, Δ*E*_2_ = −0.88 eV, Δ*E*_3_ = −1.19 eV, and Δ*E’* = −0.69 eV

On the other hand, to investigate the thermodynamic parameters and the driving force for the reduction of CO_2_, we measured the reduction potentials of the Eu_2_ SBUs and calculated the energy of {[Ru^II^(phen)_3_]}* excited state, which can provide additional insight into the photocatalytic reaction. The cyclic voltammograms (CVs) of dinuclear Eu_2_ compound shows a reversible peak at −0.69 vs. NHE (Supplementary Fig. 22). The reduction potential of Eu^III^/Eu^II^ is more negative than that of CO_2_/HCOOH (−0.58 V vs. NHE). Furthermore, the energy difference Δ*E*_1_ between excited state of {[Ru^II^(phen)_3_]}* and ground state of {[Ru^II^(phen)_3_]} can be calculated from the luminescence emission peak at 598 nm (Δ*E*_1_ = 2.07 eV, Supplementary Fig. 23a). In addition, as shown in Supplementary Fig. 23b, the CVs of H_3_**L** showed the redox potential of [Ru^III^(phen)_3_]^3+^/[Ru^II^(phen)_3_]^2+^ to be 1.19 V vs. NHE (−Δ*E*_3_). Based on the energy loop (Fig. 8), the redox potential Δ*E*_2_ of {[Ru^II^(phen)_3_]}*/[Ru^III^(phen)_3_]^3+^ was calculated to be −0.88 V vs. NHE (Δ*E*_2_ = 1.19–2.07 eV), which is more negative than the −0.69 V of the Eu_2_ SBUs to drive the reduction of Eu_2_ SBUs. These results indicate that under photocatalytic conditions, the {[Ru^II^(phen)_3_]}* transfer electrons to [Eu^III^-H_2_O-Eu^III^] unit, resulting in the reduced [Eu^II^-H_2_O-Eu^II^] unit, which then transfers electrons to CO_2_ for its reduction.

In light of the above experimental results and DFT calculations, the photocatalytic mechanism is proposed (Fig. 8). Under the visible-light irradiation, the ligand [Ru^II^(phen)_3_] will be excited to triplet MLCT excited state, {[Ru^II^(phen)_3_]}*, which can transfer one electron to the [Eu^III^-H_2_O-Eu^III^] unit through multistep relaxation and afford [Ru^III^(phen)_3_]. Consequently, the [Eu^III^-H_2_O-Eu^III^] unit accepted two electrons from two adjacent [Ru^II^(phen)_3_] to give dinuclear [Eu^II^-H_2_O-Eu^II^] active site, which can selectively reduce CO_2_ to HCOOH in a two-electron process. Finally, the [Ru^III^(phen)_3_] can be reduced back to the [Ru^II^(phen)_3_] by sacrificial donor (TEOA) to complete the catalytic cycle. Additionally, as shown in Supplementary Figs. 24–26, energy levels of the metalloligand and Eu_2_ node that are involved in the electron–hole separation process were determined with a combination of optical absorption spectra analysis and electrochemical measurements (Supplementary Note 4).

In summary, we demonstrate a photosensitizing MOF based on {Eu(III)}_2_ cluster and Ru(phen)_3_-derived ligand, featuring high photocatalytic activity for visible-light-driven CO_2_ reduction. Remarkably, the efficient electron transfer from Ru(phen)_3_-derived tricarboxylate ligand to {Eu(III)}_2_ catalytic centers allowed high formate production rate of 321.9 μmol h^−1^ mmol_MOF_^−1^. The in situ photo-generated dinuclear [Eu^II^-H_2_O-Eu^II^]-active sites are involved in two-electron reduction of CO_2_ to selectively produce HCOOH. This work not only provides a strategy to design and synthesize highly effective photocatalytic catalysts based on lanthanide cluster but also provides a platform for understanding the electron transfer mechanism in Ln-MOF materials.

## Methods

### Materials and equipment

Unless otherwise mentioned, all starting materials and chemicals were purchased from commercial vendors and used without further purification. Crystallographic data of Eu-Ru(phen)_3_-MOF was collected on a MarCCD mx300. The PRXD data were collected on Agilent SuperNova (Rigaku) with CuKα radiation sources (15 mA, 40 kV). ^1^H-NMR ^1^H-^1^H COSY and ^13^C NMR spectra were recorded on a Bruker NMR 500 DRX spectrometer at 500 MHz. The ^13^C spectra were referenced to DMSO-*d*^6^ (δ = 39.52). Mass spectrum was recorded on an Agilent Technologies ESI-MS in CH_3_CN. ICP-MS data were obtained with an Agilent 7700x ICP-MS and analyzed using ICP-MS MassHunter version B01.03. Samples were decomposed by 68% HNO_3_ and then diluted to a 2% HNO_3_ solution and analyzed with a ^159^Tb internal standard against a 10-point standard curve. The correlation coefficient was >0.9997 for all analytes of interest. TGA experiment was performed on an SDT Q600 thermal analysis system and the samples were heated from 30 to 800 °C with a slow rate of 10 °C min^−1^ under N_2_. SEM studies were performed on ZEISS SIGMA. Fluorescence spectra were taken at room temperature on Hitachi F7000 and the time-resolved PL experiments were carried out on an Edinburgh FLS980 fluorescence spectrometer. Gas sorption measurement was performed on a Micromeritics ASAP 2020 system at desired temperatures. UV–Vis studies were carried out on a UV-2401 PC UV–Vis recording spectrometer. EPR spectra were recorded on Bruker EMX-10/12 EPR spectrometer.

### Synthesis of Eu-Ru(phen)_3_-MOF

Eu(NO_3_)_3_·6H_2_O (42.0 mg, 94.2 µmol), H_3_**L** (8.0 mg, 7.1 µmol), 2-FBA (100 mg, 0.719 mmol), and DMF (3 mL) was sealed in a 20 mL Telfon-lined autoclave, and then heated to 105 °C for 70 h in a preheated oven. After cooling down to room temperature, the red block-shaped crystals (3 mg, 44% yield) were obtained. Formula of MOF: [Eu_2_(μ_2_-H_2_O)(H_2_O)_3_(L)_2_]·(NO_3_)_2_·(2-FBA)_*x*_·(H_2_O)_*y*_ (*x* ≈ 2, *Y* ≈ 22). The NO_3_^−^ counter anions, guest water molecules, and 2-FBA are severely disordered and therefore removed by SQUEEZE method using the PLATON software. The number of the NO_3_^−^ counter anions, guest water molecules, and 2-FBA were confirmed by charge balance, element analysis, and thermogravimetric analysis. Anal. Calcd. For Eu_2_Ru_2_H_126_C_134_N_26_F_2_O_48_ (FW = 3412.65, based on two 2-FBA and 22 H_2_O guest molecules): C, 47.16, H, 3.72, N, 10.67, Found: C, 47.07, H, 3.74, N, 10.35.

### Single-crystal X-ray diffraction determination of Eu-Ru(phen)_3_-MOF

Data of the Eu-Ru(phen)_3_-MOF was collected on a MarCCD mx300 at 100 K in the National Center for Protein Sciences Shanghai at the Shanghai Synchrotron Radiation Facility. Block-shaped single crystal of Eu-Ru(phen)_3_-MOF was chosen under a microscope on a plastic fiber loop for measurement. Data reduction and integration were performed with the HKL3000 software. The wavelength of X-ray corrections were performed using program of PLATON. The structures were solved by direct methods and refined to convergence by least-squares method on *F*^2^ using the SHELXTL software. The disordered guest water molecules and 2-FBA in structure of MOF were removed by using the PLATON software with SQUEEZE method. In addition, hydrogen atoms are refined isotropically. Cambridge Crystallographic Data Center (CCDC) number of 1576282 for MOF contains the supplementary crystallographic data that is summarized in Supplementary Table 5.

### Femtosecond TA spectroscopy

The TA measurements were carried out on a regenerative amplified Ti:sapphire laser system in combination with nonlinear frequency mixing techniques and the ultrafast TA spectrometer (Time-Tech Spectra, femtoTA100)^47^. During the measurements, samples under investigation were dispersed in DMF and placed in a 2 mm quartz curvette with stirring by a magnetic stirrer, constantly. Detailed procedures for the femtosecond TA measurements can be found in the Supplementary Methods.

### Photocatalytic reactions

The photocatalytic activities of the samples were performed via a controllable reaction system (CEL-SPH2N, CEAULight, China) with a volume of approximately 300 mL. The setup of the photocatalytic system is shown in Supplementary Fig. 12. The mixture of catalyst MOF 50 mg or H_3_**L** 50 mg, TEOA (2.0 mL), and CH_3_CN (40.0 mL) was sealed in a 100 mL Pyrex flask. To remove the dissolved oxygen, the flask was capped with a quartz septum and degassed with a pure CO_2_ for 30 min. The light source is a 300 W xenon lamp through a UV cut filter with a wavelength range of 420–800 nm. The HCOO^−^ in liquid phase was quantified by an ion chromatography (881 Compact IC pro, Metrosep) with a Metrosep A supp 5 250/4.0 column under 303 K. In addition, the eluent is the mixed solution of NaHCO_3_ (1000 μM) and Na_2_CO_3_ (3200 μM). Gas productions were performed on a gas chromatograph (Aulight GC-7920) equipped with a thermal conductivity detector (TCD) and flame ionization detector (FID). After each reaction time, the evolved gaseous phase in the headspace of the Pyrex flask was sampled with a gastight syringe (500 µL) and measured by GC (N_2_ as a carrier gas) using the TCD (a packed column with molecular sieves 5 A (3.0 m × 3.0 mm, 60–80 mesh) at 373 K) to detect H_2_ and using the FID (a column (TDX-1, 3.0 m × 0.30 mm) at 653 K) to detect CH_4_ and CO. However, no signals for H_2_, CH_4_, and CO can be observed.

### In situ EPR experiments

The in situ EPR experimental data were obtained over a commercial EPR spectrometer Bruker EMX-10/12 at a X-band (9 GHz). Samples were prepared by mixing the catalyst in a glass tube with 0.5 mL solution of CH_3_CN/TEOA (20:1). The sample was degassed by N_2_ and then sealed. Then the glass tube was fixed into the EPR resonator. When needed, the CO_2_ were introduced into the sample. The experiments were performed under visible-light irradiation.

### Data availability

The X-ray crystallographic data for structure Eu-Ru(phen)_3_-MOF has been deposited at the CCDC, with a CCDC number of 1576282. The data can be obtained free of charge from The Cambridge Crystallographic Data Centre via www.ccdc.cam.ac.uk/data_request/cif. All other data supporting the findings of this study are available within the article and its Supplementary Information files, or from the corresponding author on reasonable request.

## Electronic supplementary material

Supplementary Information

## References

[1] Sanz-Pérez ES, Murdock CR, Didas SA, Jones CW (2016). Direct capture of CO_2_ from ambient air. Chem. Rev..

[2] Schiermeier Q (2011). Increased flood risk linked to global warming: likelihood of extreme rainfall may have been doubled by rising greenhouse-gas levels. Nature.

[3] Xu H (2015). Visible-light photoreduction of CO_2_ in a metal-organic framework: boosting electron–hole separation via electron trap states. J. Am. Chem. Soc..

[4] Zhang H (2016). Surface-plasmon-enhanced photodriven CO_2_ reduction catalyzed by metal-organic-framework-derived iron nanoparticles encapsulated by ultrathin carbon layers. Adv. Mater..

[5] Wang S, Yao W, Lin J, Ding Z, Wang X (2014). Cobalt imidazolate metal-organic frameworks photosplit CO_2_ under mild reaction conditions. Angew. Chem. Int. Ed..

[6] Inoue T, Fujishima A, Konishi S, Honda K (1979). Photoelectrocatalytic reduction of carbon dioxide in aqueous suspensions of semiconductor powders. Nature.

[7] Inoue H, Moriwaki H, Maeda K, Yoneyama H (1995). Photoreduction of carbon dioxide using chalcogenide semiconductor microcrystals. Photochem. Photobiol. A.

[8] Fujiwara H (1997). Effect of surface structures on photocatalytic CO_2_ reduction using quantized CdS nanocrystallites. J. Phys. Chem. B.

[9] Yan SC (2010). A room-temperature reactive-template route to mesoporous ZnGa_2_O_4_ with improved photocatalytic activity in reduction of CO_2_. Angew. Chem. Int. Ed..

[10] Duan L, Wang L, Li F, Sun L (2015). Highly efficient bioinspired molecular Ru water oxidation catalysts with negatively charged backbone ligands. Acc. Chem. Res..

[11] Long LL (2016). Layer-controlled growth of MoS_2_ on self-assembled flower-like Bi_2_S_3_ for enhanced photocatalysis under visible light irradiation. NPG Asia Mater..

[12] Yamamoto M (2016). Visible light-driven water oxidation using a covalently-linked molecular catalyst-sensitizer dyad assembled on a TiO_2_ electrode. Chem. Sci..

[13] Li H (2015). Visible light-driven water oxidation promoted by host–guest interaction between photosensitizer and catalyst with a high quantum efficiency. J. Am. Chem. Soc..

[14] Schoedel A, Li M, Li D, O’Keeffe M, Yaghi OM (2016). Structures of metal-organic frameworks with rod secondary building units. Chem. Rev..

[15] Zhou HC, Kitagawa S (2014). Metal–organic frameworks (MOFs). Chem. Soc. Rev..

[16] Cook TR, Zheng YR, Stang PJ (2013). Metal-organic frameworks and self-assembled supramolecular coordination complexes: comparing and contrasting the design, synthesis, and functionality of metal-organic materials. Chem. Rev..

[17] Xuan W, Zhu CF, Liu Y, Cui Y (2014). Mesoporous metal-organic framework materials. Chem. Soc. Rev..

[18] Trickett CA (2017). The chemistry of metal-organic frameworks for CO_2_ capture, regeneration and conversion. Nat. Rev. Mater..

[19] Fu Y (2012). An amine-functionalized titanium metal-organic framework photocatalyst with visible-light-induced activity for CO_2_ reduction. Angew. Chem. Int. Ed..

[20] Sun D (2013). Studies on photocatalytic CO_2_ reduction over NH_2_-Uio-66 (Zr) and its derivatives: towards a better understanding of photocatalysis on metal-organic frameworks. Chem. Eur. J..

[21] Zhang H (2016). Efficient visible-light-driven carbon dioxide reduction by a single-atom implanted metal–organic framework. Angew. Chem. Int. Ed..

[22] Vincent, K. A. et al. A. Instantaneous, stoichiometric generation of powerfully reducing states of protein active sites using Eu(II) and polyaminocarboxylate ligands. *Chem. Commun*. **0**, 2590–2591 (2003). 10.1039/b308188e 14594295

[23] Lee CC, Hu Y, Ribbe MW (2012). ATP-independent substrate reduction by nitrogenase P-cluster variant. Proc. Natl. Acad. Sci. USA.

[24] Zhang S, Li L, Zhao S, Sun Z, Luo J (2015). Construction of interpenetrated ruthenium metal-organic frameworks as stable photocatalysts for CO_2_ reduction. Inorg. Chem..

[25] Kajiwara T (2016). Photochemical reduction of low concentrations of CO_2_ in a porous coordination polymer with a ruthenium(II)–CO complex. Angew. Chem. Int. Ed..

[26] Wang C, Xie Z, de Krafft EK, Lin W (2011). Doping metal-organic frameworks for water oxidation, carbon dioxide reduction, and organic photocatalysis. J. Am. Chem. Soc..

[27] Alvaro M (2017). Semiconductor behavior of a metal-organic framework (MOF). Chem. Eur. J..

[28] Tachikawa T, Choi JR, Fujitsuka M, Majima T (2008). Photoinduced charge-transfer processes on MOF-5 nanoparticles: elucidating differences between metal-organic frameworks and semiconductor metal oxides. J. Phys. Chem. C.

[29] Spek AL (2003). Single-crystal structure validation with the program PLATON. J. Appl. Crystallogr..

[30] Ma L, Falkowski JM, Abney C, Lin W (2010). A series of isoreticular chiral metal-organic frameworks as a tunable platform for asymmetric catalysis. Nat. Chem..

[31] Ferey G, Mellot-Draznieks C, Serre C, Millange F (2005). Crystallized frameworks with giant pores: are there limits to the possible?. Acc. Chem. Res..

[32] Fan J (2011). Synergistic effect of N and Ni^2+^ on nanotitania in photocatalytic reduction of CO_2_. J. Environ. Eng..

[33] Zhang Q, Li Y, Ackerman EA, Gajdardziska-Josifovska M, Li H (2011). Visible light responsive iodine-doped TiO_2_ for photocatalytic reduction of CO_2_ to fuels. Appl. Catal. A.

[34] Wang JL, Wang C, Lin W (2012). Metal–organic frameworks for light harvesting and photocatalysis. ACS Catal..

[35] Pan Q (2014). Directionality of ultrafast electron transfer in a hydrogen evolving Ru-Pd-based photocatalyst. J. Phys. Chem. C.

[36] Karnahl M (2011). Tuning of photocatalytic hydrogen production and photoinduced intramolecular electron transfer rates by regioselective bridging ligand substitution. Chem. Phys. Chem..

[37] Li R (2014). Integration of an inorganic semiconductor with a metal-organic framework: a platform for enhanced gaseous photocatalytic reactions. Adv. Mater..

[38] Wu K, Zhu H, Liu Z, Rodríguez-Córdoba W, Lian T (2012). Ultrafast charge separation and long-lived charge separated state in photocatalytic CdS-Pt nanorod heterostructures. J. Am. Chem. Soc..

[39] Yang S, Pattengale B, Lee S, Huang J (2018). Real-time visualization of active species in a single-site metal-organic framework photocatalyst. ACS Energy Lett..

[40] Chen S (2016). A metal-organic cage incorporating multiple light harvesting and catalytic centres for photochemical hydrogen production. Nat. Commun..

[41] Havlák L (2016). Eu^2+^ stabilization in YAG structure: optical and electron paramagnetic resonance study. J. Phys. Chem. C.

[42] Dehelean A, Rada S, Popa A, Suciu RC, Culea E (2016). Raman, photoluminescence and EPR spectroscopic characterization of europium(III) oxide-lead dioxide-tellurite glassy network. J. Lumin..

[43] Kresse G, Furthmüller J (1996). Efficient iterative schemes for *ab initio* total-energy calculations using a plane-wave basis set. Phys. Rev. B..

[44] Anisimo VI, Aryasetiawan F, Lichtenstein IA (1997). First-principles calculations of the electronic structure and spectra of strongly correlated systems: the LDA+ U method. J. Phys. Condens. Matter.

[45] Yu M, Trinkle DR (2011). Accurate and efficient algorithm for Bader charge integration. J. Chem. Phys..

[46] Zheng ZG, Lin CZ, Chen QY (2008). Di*-µ*-methacrylato-bis[diaquabis(methacrylato)europium(III)] methacrylic acid disolvate. Acta Crystallogr..

[47] Liu, J., Leng, J., Wu, K., Zhang, J. & Jin, S. Observation of internal photoinduced electron and hole separation in hybrid two-dimentional perovskite films. *J. Am. Chem. Soc*. **139**, 1432–1435 (2017). 10.1021/jacs.6b12581 28094931

