# Supplementary material for: Photo-generated dinuclear {Eu(II)}2 active sites for selective CO2 reduction in a photosensitizing metal-organic framework
Source: Nat Commun. 2018 Aug 22;9:3353. doi: 10.1038/s41467-018-05659-7 (PMC6105582; doi:10.1038/s41467-018-05659-7)
Supplement: Supplementary file 1 — Supplementary Information [file 41467_2018_5659_MOESM1_ESM.pdf]

Supplementary Information for

Photo-generated Dinuclear {Eu(II)}$_2$ Active Sites for Selective CO_2 Reduction in a Photosensitizing Metal−Organic Framework

Zhi-Hao Yan *et al*

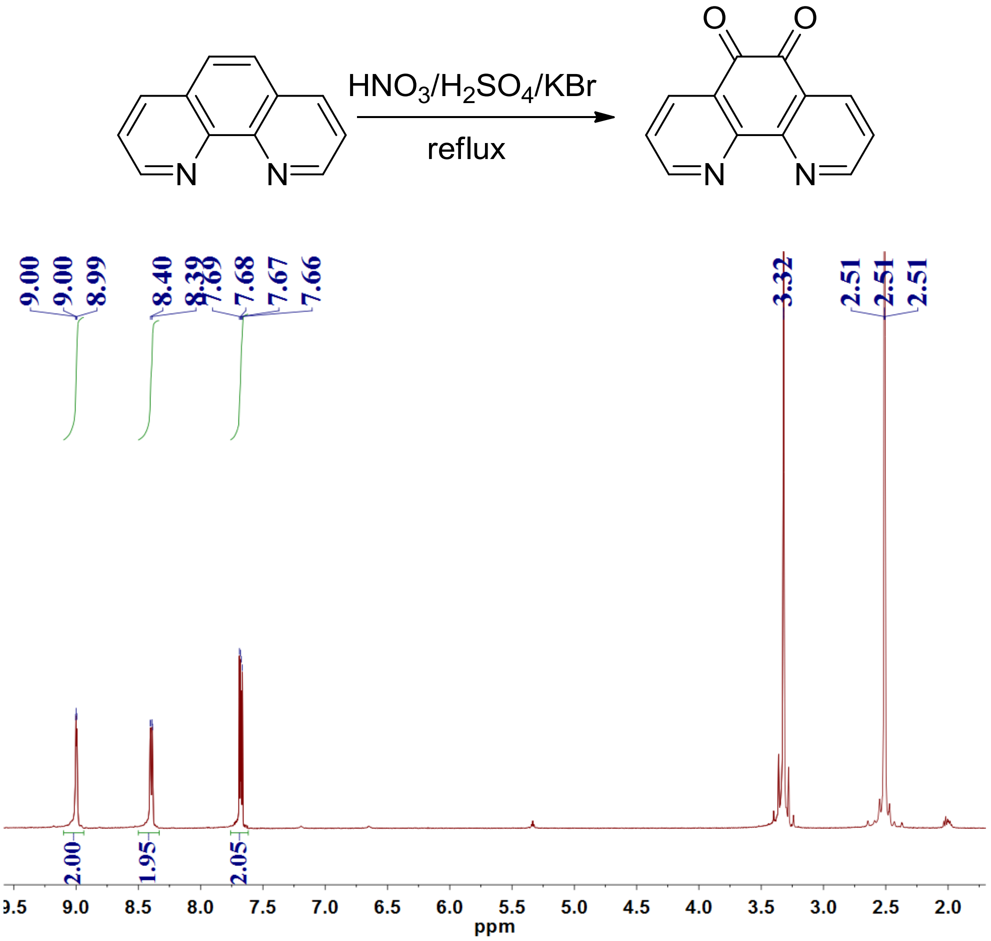

Supplementary Figure 1. ^{1}H-NMR spectrum (500MHz) of 1,10-Phenanthroline-5,6-dione in DMSO-d_6.

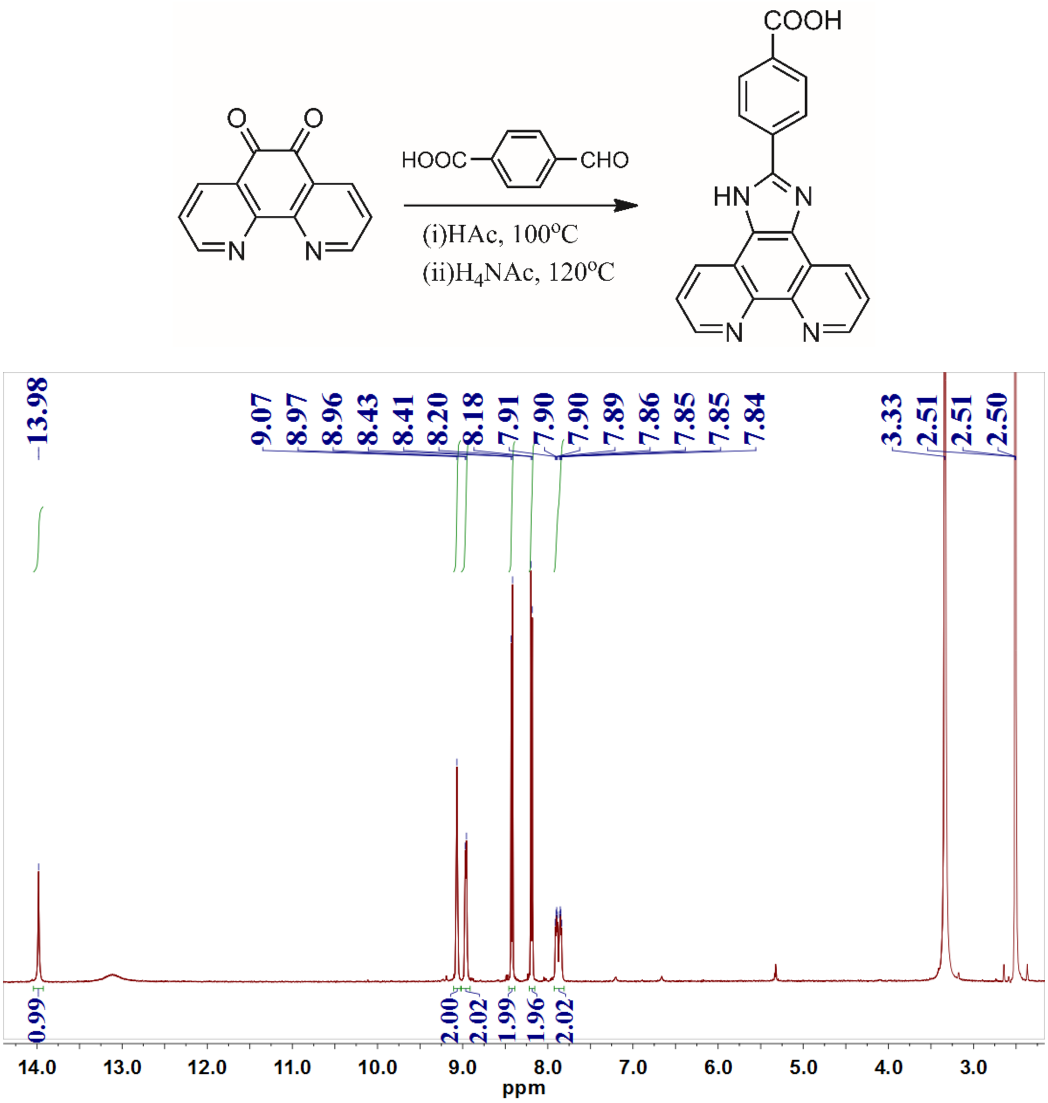

Supplementary Figure 2. ^{1}H-NMR spectrum of 4-(1*H*-imidazo[4,5-*f*][1,10]phenanthrolin-2-yl) benzoic acid in DMSO-d_6.

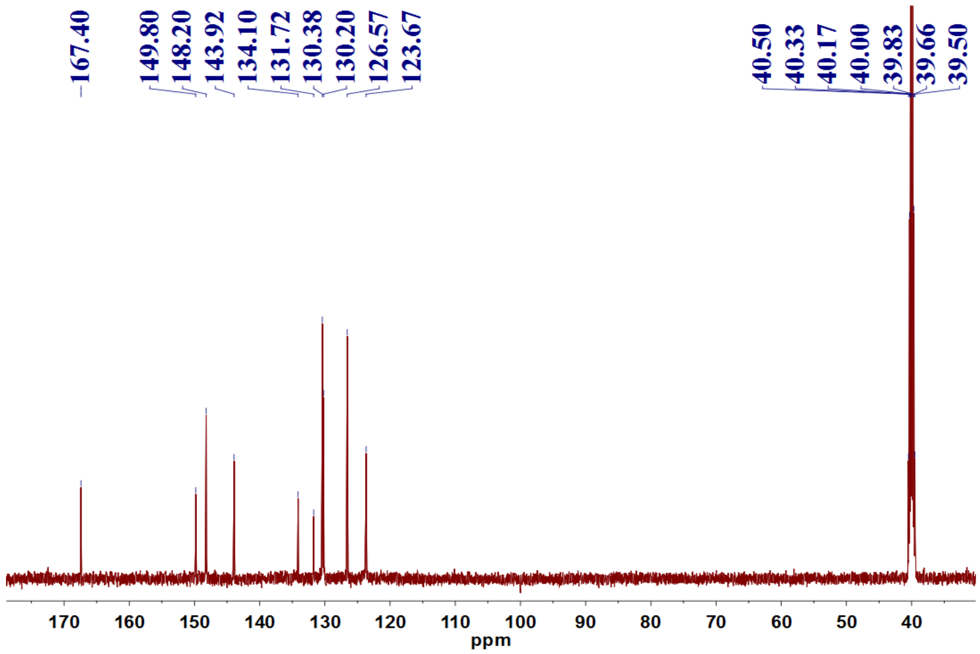

Supplementary Figure 3. ^{13}C-NMR spectrum of 4-(1*H*-imidazo[4,5-*f*][1,10]phenanthrolin-2-yl) benzoic acid in DMSO-d_6.

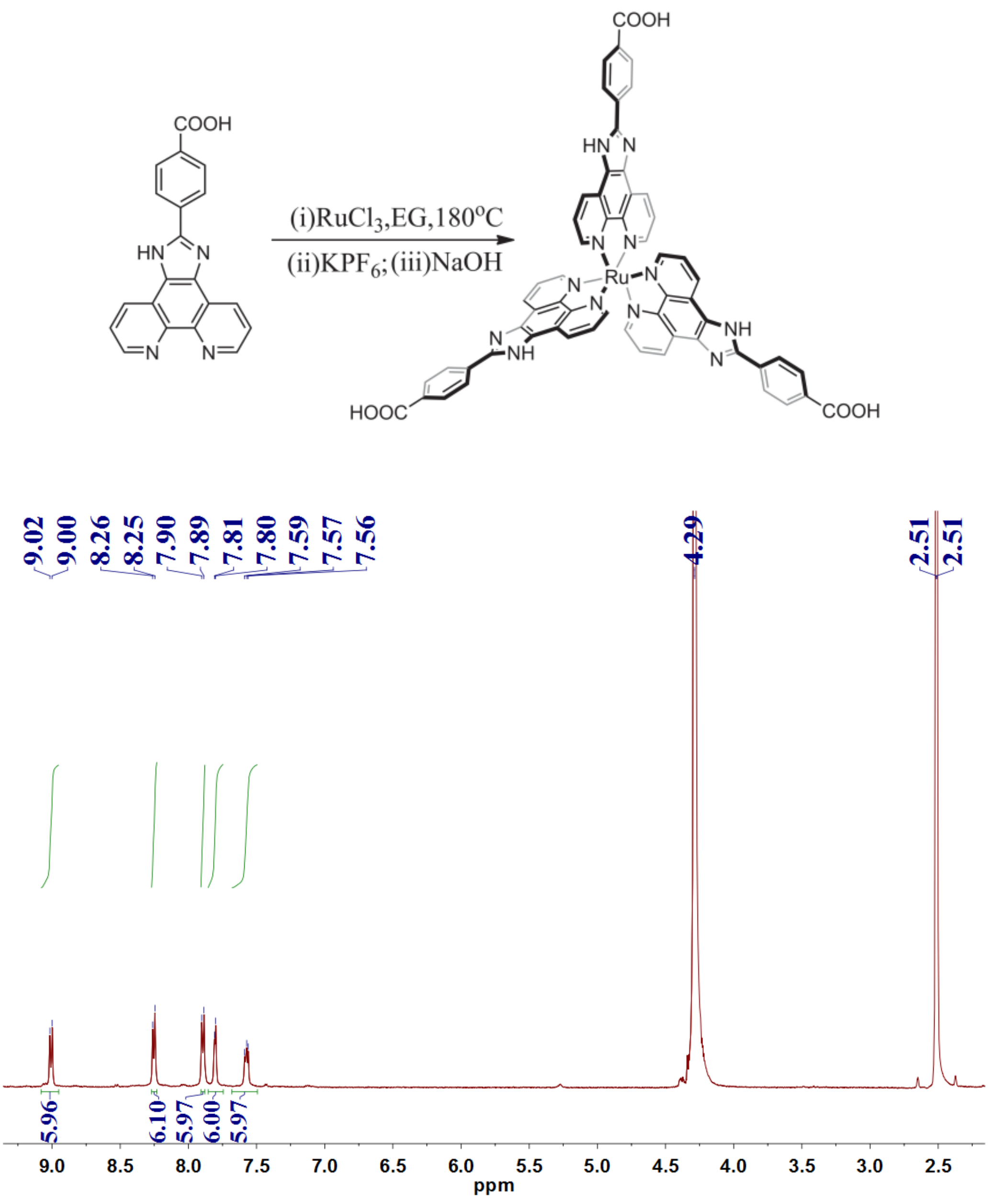

Supplementary Figure 4. ^{1}H-NMR spectrum (500 MHz) of H$_3$**L** in d_6-DMSO-D$_2$O (v:v=4:1).

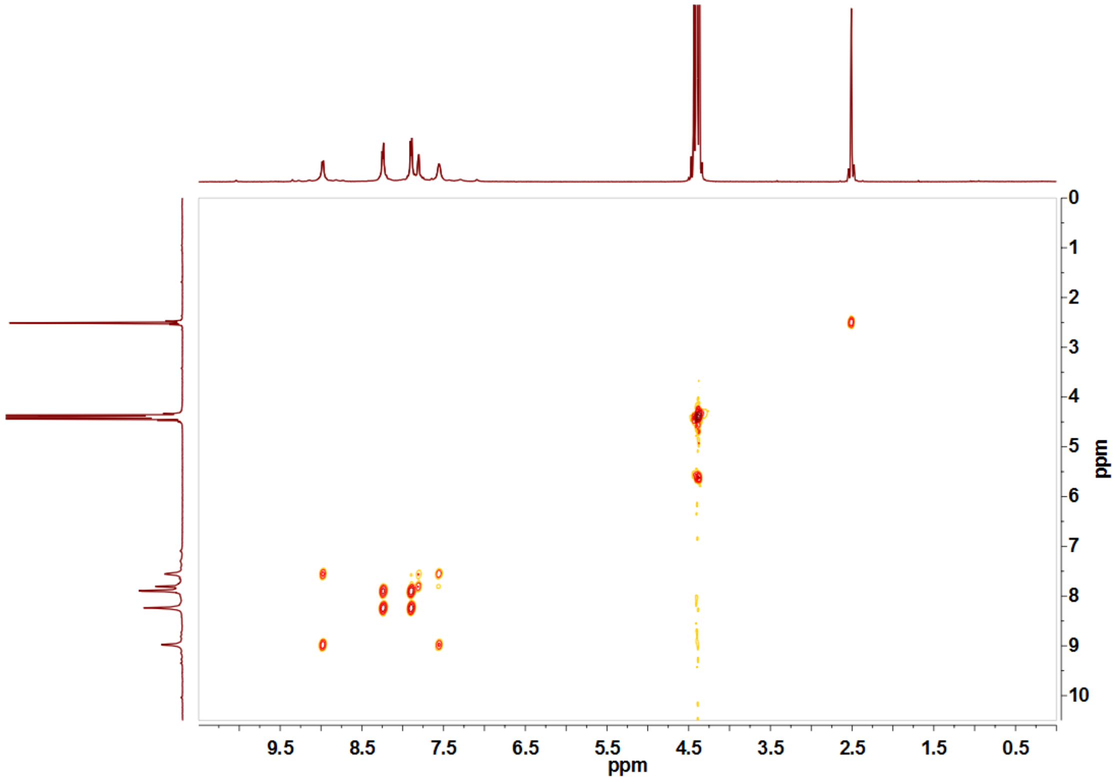

Supplementary Figure 5. ^{1}H-^{1}H-COSY spectrum (500 MHz) of H_3**L** in d_6-DMSO-D_2O (v:v=4:1).

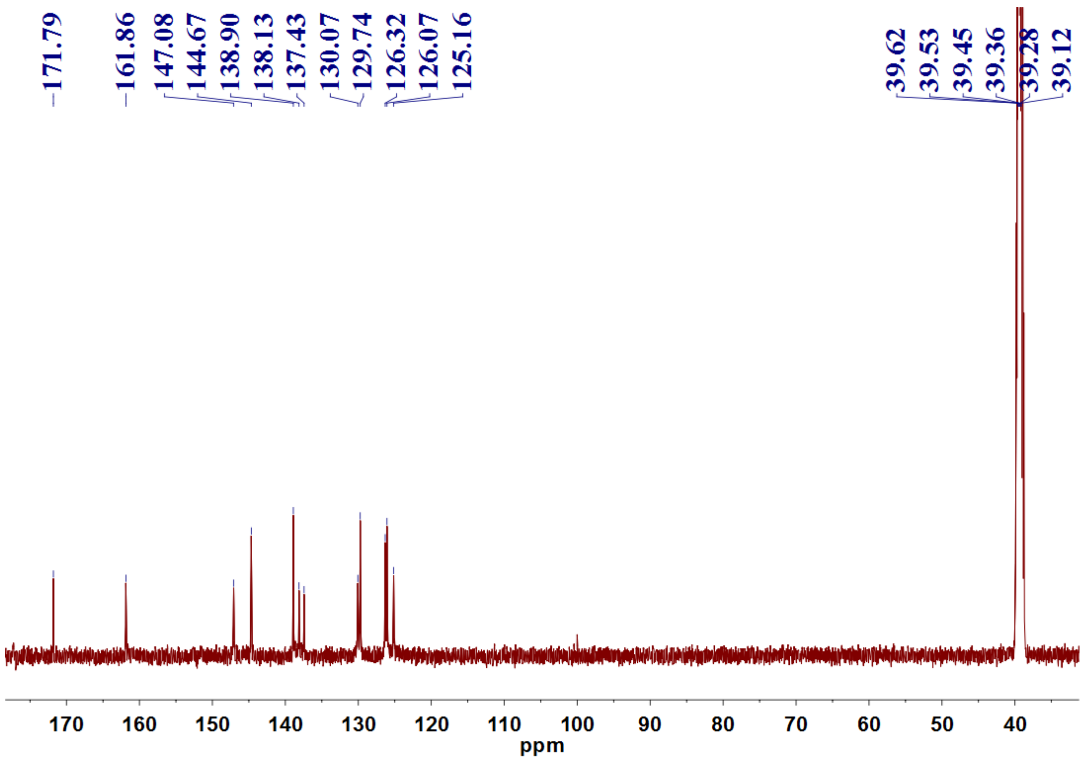

Supplementary Figure 6. ^{13}C-NMR spectrum of H_3**L** in d_6-DMSO-D_2O (*v*:*v*=4:1).

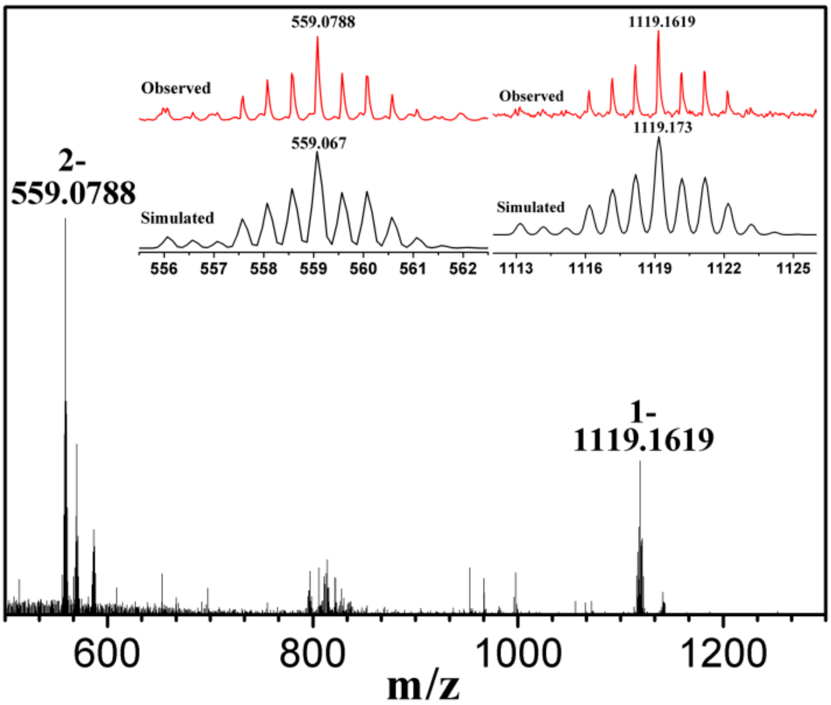

Supplementary Figure 7. ESI-MS spectra of the H_3L. The sequence of peaks with insets showing the observed and simulated isotope patterns of the 2- and 1- peaks.

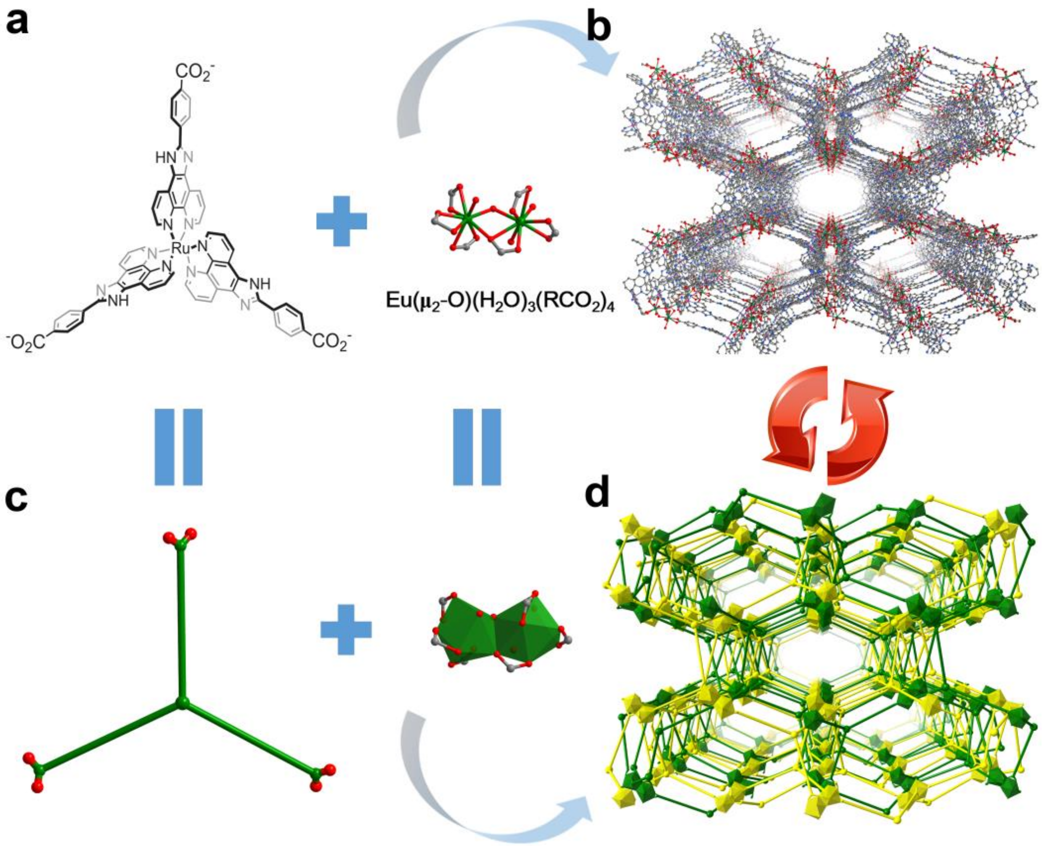

Supplementary Figure 8. Crystal structure of Eu-Ru(phen)$_3$-MOF. a Chemical Structures of H_3**L** and Eu_2 cluster SBUs. **b** The crystal structure of Eu-Ru(phen)$_3$-MOF viewed along the (100) direction. **c** Ball-and-stick model showing the H_3**L** with D_3 symmetry. **d** 2-fold interpenetrated Eu-Ru(phen)$_3$-MOF; Yellow color indicates the interpenetrated framework.

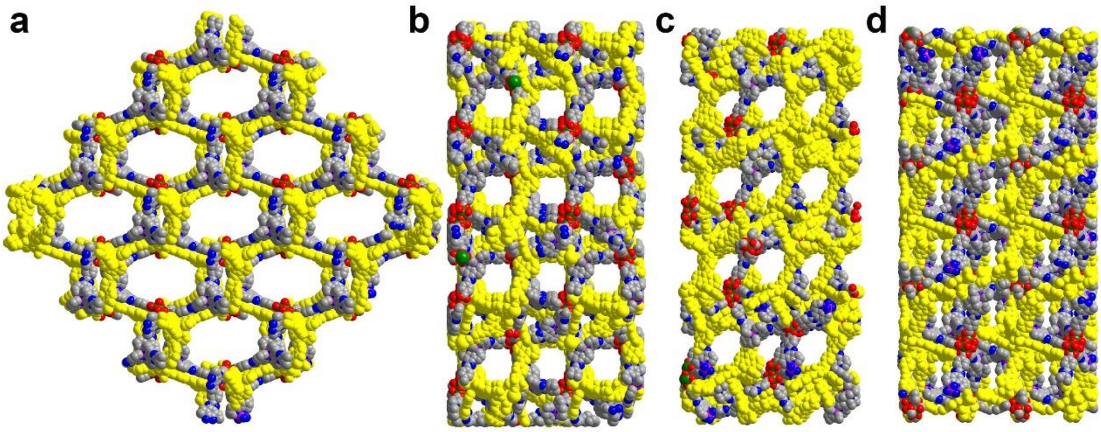

Supplementary Figure 9. Space-filling model of Eu-Ru(phen)$_3$-MOF. a Viewed along the (100) direction, the channel sizes are 16 Å×31 Å. **b** Viewed along the (011) direction, the channel sizes are 15Å×20 Å. **c** Viewed along the (111) direction, the channel sizes are 14 Å×15 Å and **d** Viewed along the (010) direction.

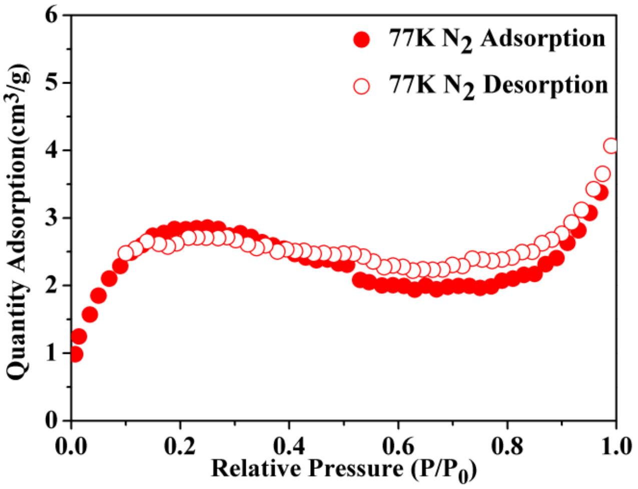

Supplementary Figure 10. Nitrogen sorption isotherms for Eu-Ru(phen)$_3$-MOF at 77 K.

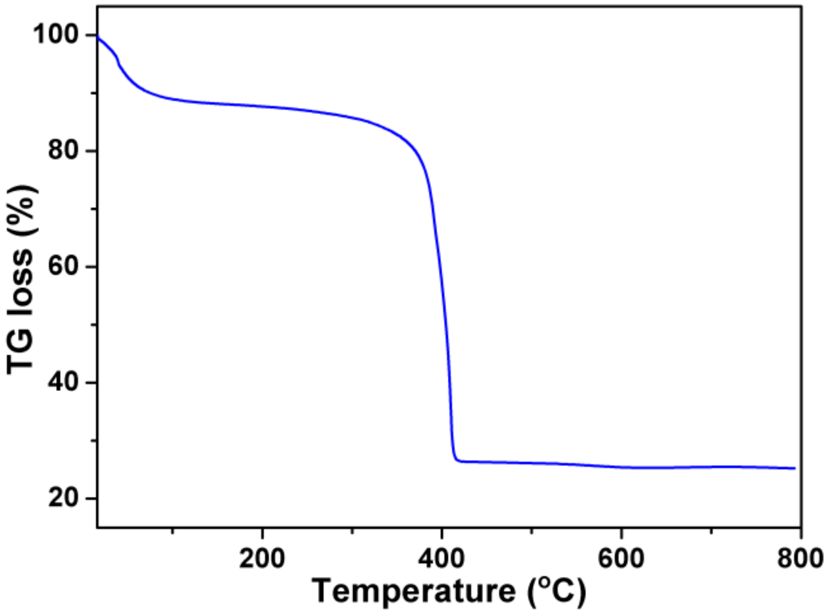

Supplementary Figure 11. TGA of Eu-Ru(phen)$_3$-MOF. The sample was heated to 800 ^{o}C under N_2 at a heating rate of 10 ^{o}C min^{-1}. A solvent weight loss of 12.3% was observed from room temperature to 200 ^{o}C.

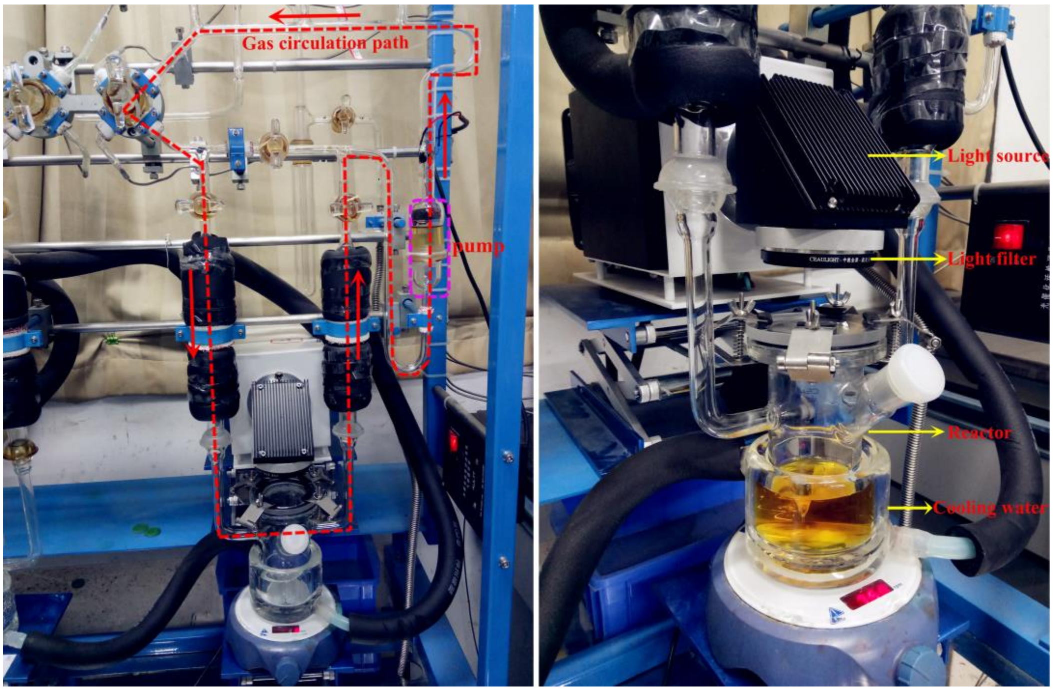

Supplementary Figure 12. A closed gas circulation and visible light induced system for CO_2 reduction.

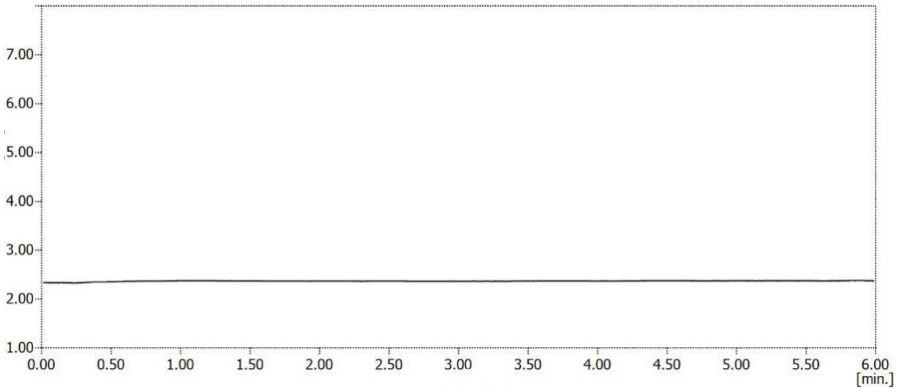

Supplementary Figure 13. GC analysis of the gaseous reaction products by using the TCD.

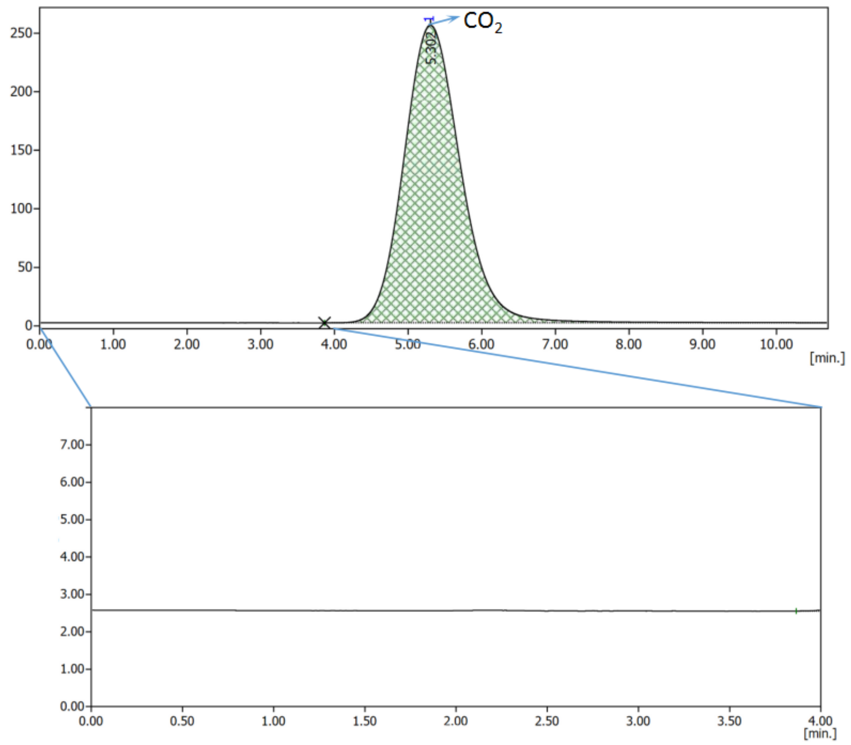

Supplementary Figure 14. GC analysis of the gaseous reaction products by using the FID.

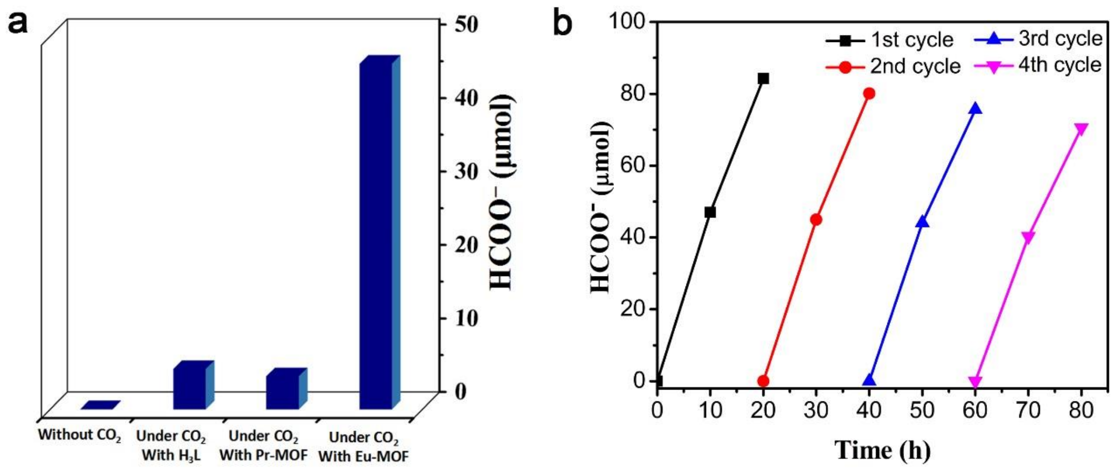

Supplementary Figure 15. The activity and stability of catalyst. a Product obtained from the photocatalytic reaction of irradiation (420 nm < λ < 800 nm) in CH_3CN/TEOA (20:1 *v/v*) solutions at 10 h without CO_2, or with H_3L under CO_2 or with Pr-Ru(phen)$_3$-MOF under CO_2 and with Eu-Ru(phen)$_3$-MOF under CO_2 atmosphere. In the absence of CO_2, almost no generation of $HCOO^-$ was observed in the presence of Eu-Ru(phen)$_3$-MOF after 10 h irradiation, supporting that $HCOO^-$ is derived exclusively from CO_2 together with the ^{13}C-labeling experiments as mentioned above. Moreover, the negligible amount of $HCOO^-$ produced with Pr-Ru(phen)$_3$-MOF under CO_2 can be attributed to that there were no valence changes in the Pr oxo-clusters. **b** The photocatalytic stability of Eu-Ru(phen)$_3$-MOF.

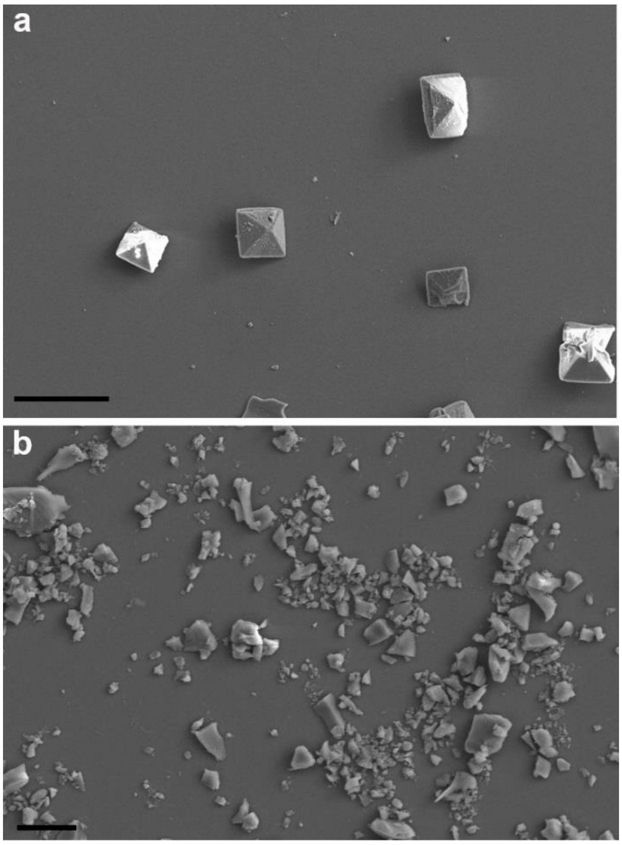

Supplementary Figure 16. Morphology of the Eu-Ru(phen)$_3$-MOF. a The SEM image of Eu-Ru(phen)$_3$-MOF before catalysis. Scale bar, 100 μm. **b** The SEM image of Eu-Ru(phen)$_3$-MOF after catalysis. Scale bar, 20 μm.

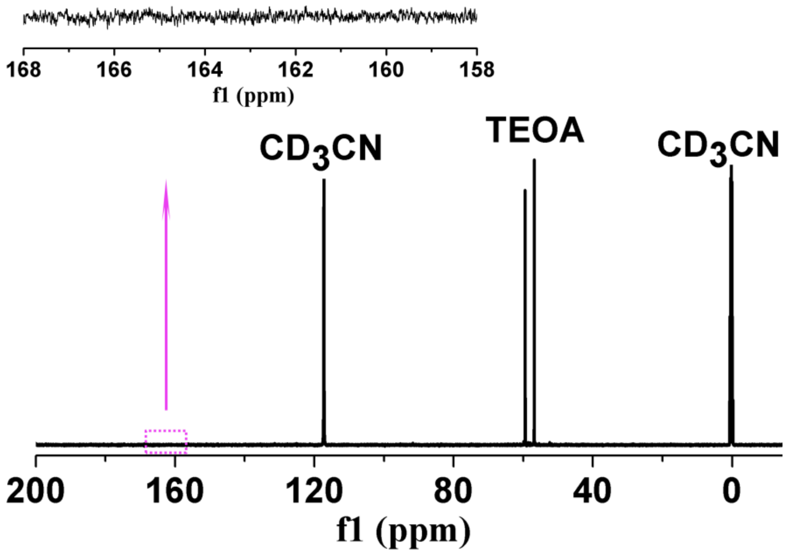

Supplementary Figure 17. The ^{13}C NMR spectrum for the product obtained from the reaction with $^{12}CO_2$ in mixture of CD_3CN/TEOA (20:1 *v*/*v*) under visible light. No peak can be assigned to be $HCOO^-$ species.

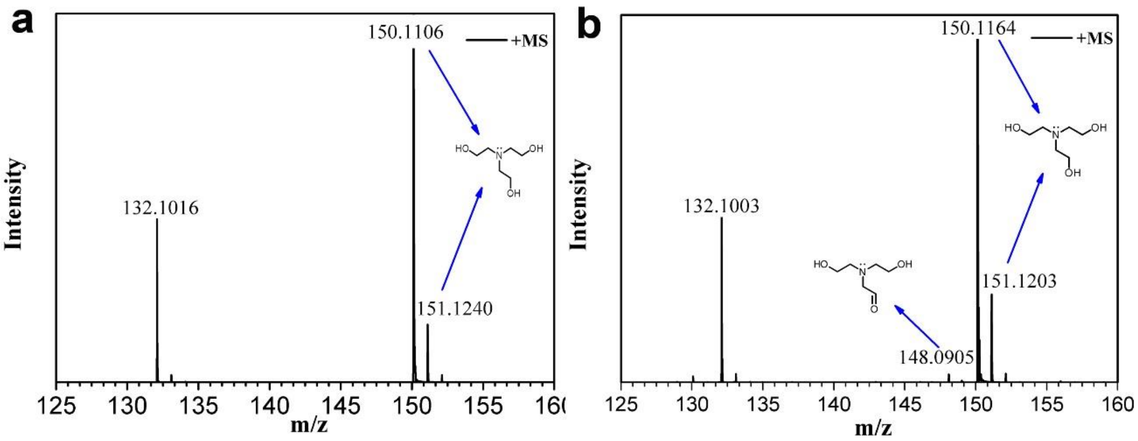

Supplementary Figure 18. ESI-MS spectra for TEOA. a before reaction. **b** after reaction.

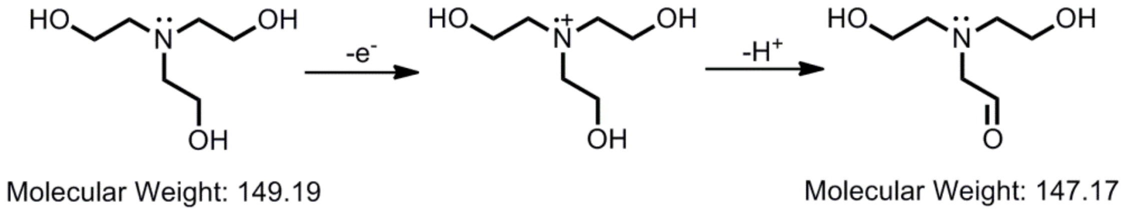

Supplementary Figure 19. Proposed TEOA degradation during the process of photocatalysis.

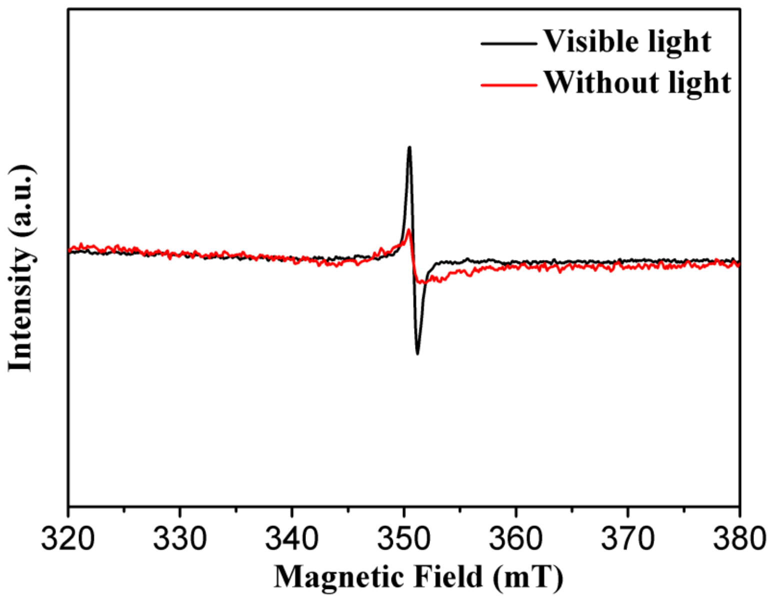

Supplementary Figure 20. EPR spectra of the H_3**L** ligand in the dark or under visible light.

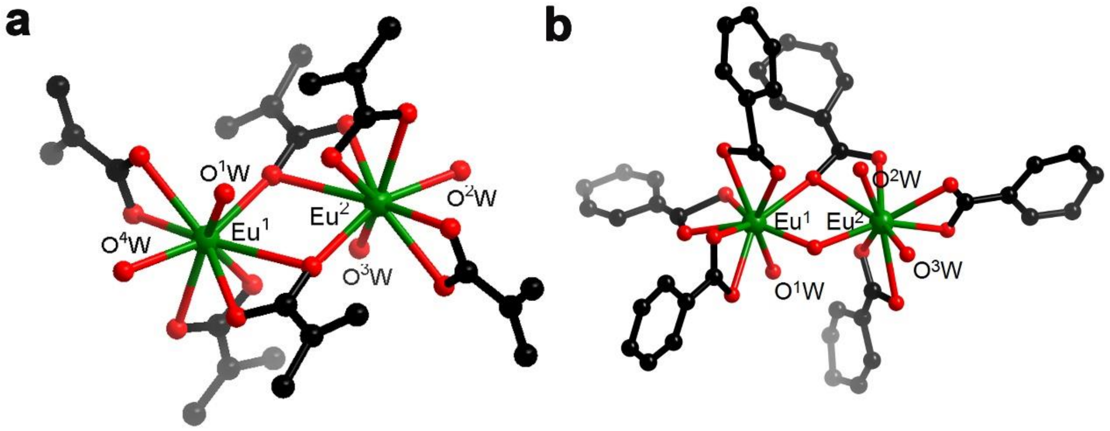

Supplementary Figure 21. a Chemical Structures of [$Eu_2(MMA)_6(H_2O)_4$]. **b** Eu_2 SBUs in Eu-Ru(phen)$_3$-MOF.

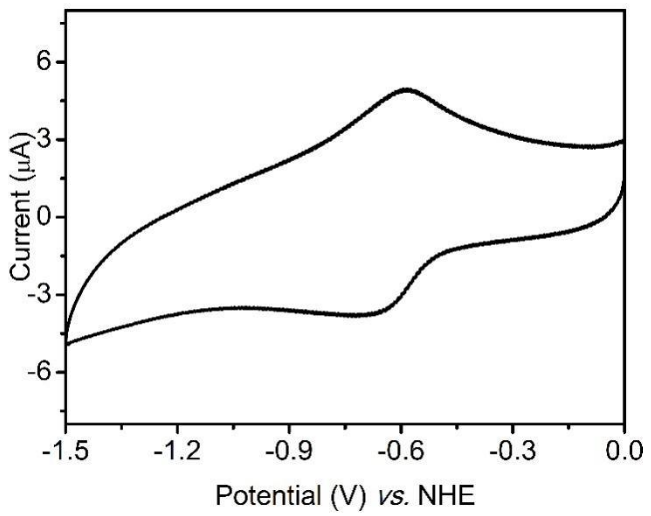

Supplementary Figure 22. CVs of $[Eu_2(MMA)_6(H_2O)_4]$ in DMF containing 0.10 M of $TBAPF_6$ (TBA = tetra-*n*-butylammonium) under Ar at a scan rate of 500 mV s^{-1}.

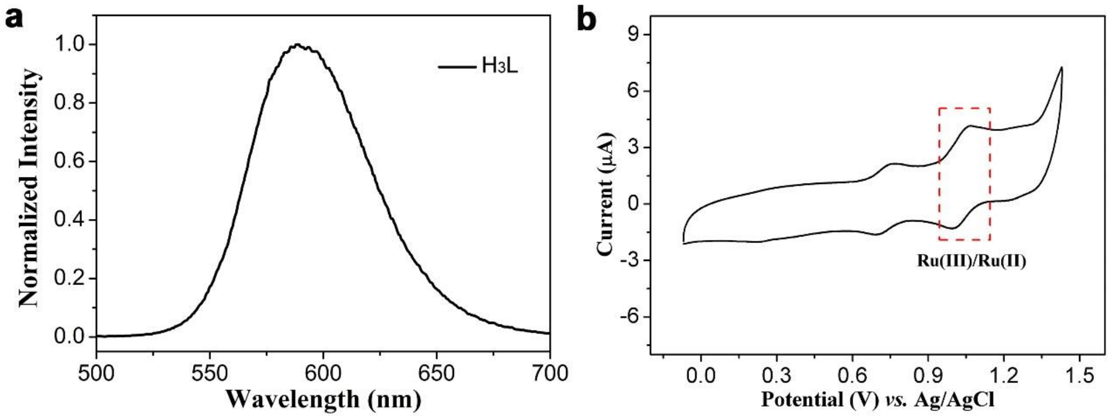

Supplementary Figure 23. Spectroscopic and electrochemical properties of H_3L. a Emission spectra of H_3**L** (λ_{ex} = 465 nm). **b** Cyclic voltamograms of 1mM H_3**L** at a scan rate of 50 mV s^{-1} in a 0.1 M $(Bu_4N)PF_6$ DMSO solution under an CO_2 atmosphere.

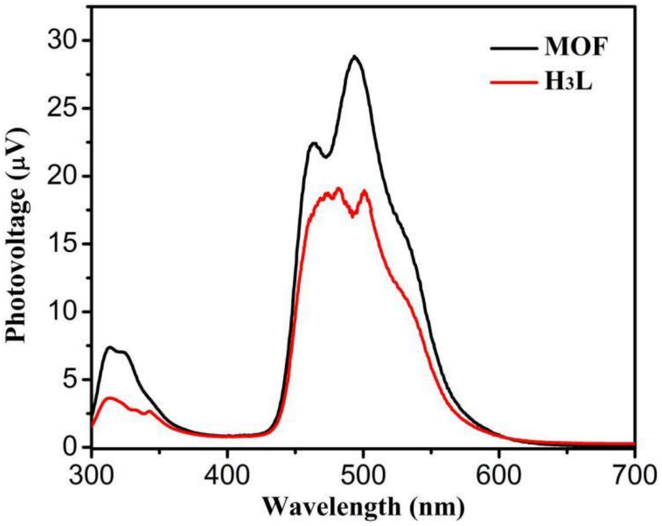

Supplementary Figure 24. SPV spectra of the Eu-Ru(phen)$_3$-MOF and H$_3$L. In the SPV spectra, both metalloligand and MOF present obvious positive photovoltage responses when irradiated by light ranging from 300 to 800 nm. MOF displays a widerange SPV response from 450 nm to 550 nm, indicating its visible-light-induced charge generation. Moreover, the signal intensity for MOF is higher than that metalloligand, suggesting a higher separation efficiency of photogenerated charge for MOF.

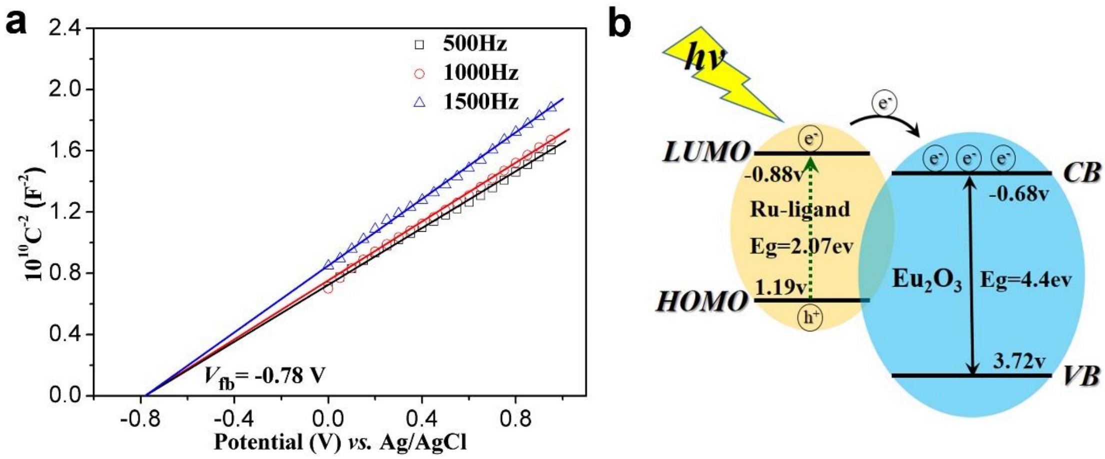

Supplementary Figure 25. Energy levels of the Eu_2O_3 and H_3L. a Mott-Schottky plot of Eu_2O_3 in 0.2 M Na_2SO_4 aqueous solution at frequency of 0.5kHz, 1.0 kHz and 1.5kHz. **b** The energy diagram of the LUMO and HOMO levels of H_3**L** and the CB and VB levels of Eu_2O_3, as well as supported the charge separation and transfer processes in the composite photocatalyst. (*VB* = valence band, *CB* = conduction band).

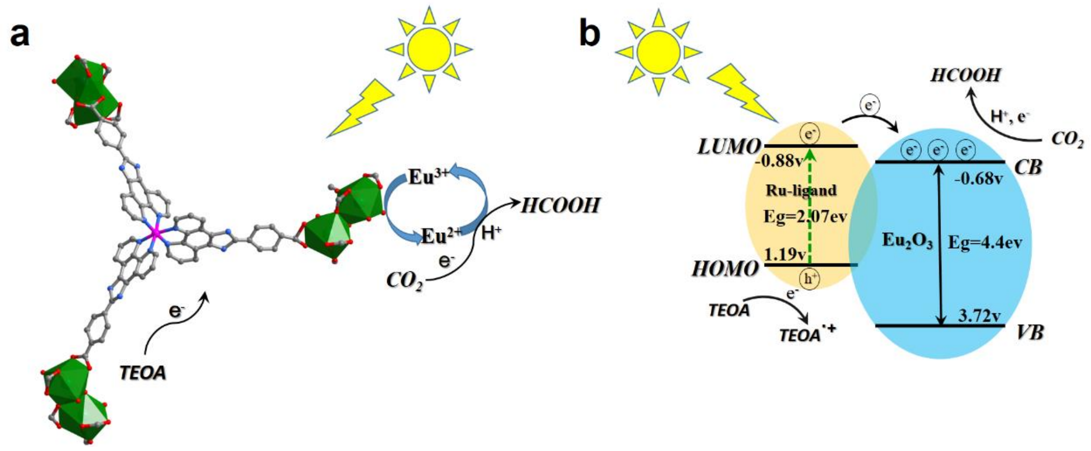

Supplementary Figure 26. Proposed catalytic mechanism. a Mechanisms underlying the photoexcited dynamics involved in Eu-(phen)$_3$-MOF and the possible reaction pathways for CO_2 conversion. **b** The energy level diagram in Eu-(phen)$_3$-MOF.

Supplementary Table 1. Comparation of the various photocatalytic performances of MOFs for CO_2 photoreduction to formate

Catalyst	Rate of $HCOO^-$ [μmol $h^{-1}mmol_{MOF}^{-1}$] [a]	TON	TOF	Catalytic sites	Constitution [b]	Ref.
NH_2-MIL-125(Ti)	26.5	0.26	0.026	Ti-O cluster	$Ti_8O_8(OH)_4(L^1)_6$	*Angew. Chem.* **124**, 3420 (2012)
NH_2-UiO-66(Zr)	46.3	0.46	0.046	Zr-O cluster	$Zr_6O_4(OH)_4(L^1)_6$	*Chem.-Eur. J.* **19**, 14279 (2013)
MIL-101(Fe)	66.7	0.53	0.067	Fe-O cluster	$Fe_3O(OH_2)_3L^1Cl$	*ACS Catal.* **4**, 4254 (2014)
Mixed H_2N-UIO-66(Zr)	73.4	0.73	0.073	Zr-O cluster	$Zr_6O_4(OH)_4(L^1)_{4.8}(L^2)_{1.2}$	*Chem.-Eur. J.* **19**, 14279 (2013)
Zr-SDCA-NH_2	96.2	1.15	0.096	Zr-O cluster	$[Zr_6O_4(OH)_4(L^4)_6$	*Dalton Trans.* **47**, 909 (2018)
NH_2-MIL-101(Fe)	136.3	1.09	0.136	Fe-O cluster	$Fe(OH)(L^1)$ $0.3L^1$	*ACS Catal.* **4**, 4254 (2014)
PCN-222	143.5	1.43	0.143	Zr-O cluster	$Zr_6(\mu_3\text{-}OH)_8(OH)_8(TCPP)_2$	*J. Am. Chem. Soc.* **137**, 13440 (2015)
NNU-28	183.3	1.83	0.183	Zr-O cluster	$Zr_6O_4(OH)_4(L^3)_6$	*J. Mater. Chem. A* **4**, 2657 (2016)
Ir-CP	251.7	1.51	0.251	Ir-ligand	$Y[Ir(ppy)_2(dcbpy)]_2[OH]$	*Chem. Sci.* **5**,3808 (2014)
Eu-Ru(phen)$_3$-MOF	321.9	3.22	0.322	Eu-O cluster	$[Eu_2(\mu_2\text{-}H_2O)(H_2O)_3(L)_2]$ $(NO_3)_2$ (2-$FBA^-)_2$ $(H_2O)_{22}$	This work

a: 1 mmol MOF is equivalent to 1 mmol Catalytic sites.

b: H_2L^1=2-aminoterephthalic acid, H_2L^2=2,5-diaminoterephthalic acid, H_2L^3= 4,4'-(anthracene-9,10-diylbis(ethyne-2,1-diyl))dibenzoic acid, H_2L^4 = 2,2'-diamino-4,4'-stilbenedicarboxylic acid, H_3L = Ru-L metalloligand

Supplementary Table 2. The bi-exponential fitting parameters of the PL decay traces of Eu-Ru(phen)$_3$-MOF and H_3L in the 50 ns time window shown in Fig. 4b (λ_{ex} = 377 nm)

	A_1	τ_1/ns	A_2	τ_2/ns
H_3**L**	236.932	1.557	156.935	51.34
Eu-Ru(phen)$_3$-MOF	1779.672	0.9891	856.576	12.47

The electron transfer time (t^1_{LMCT}) was found to be 6.1 ns.

Supplementary Table 3. The bi-exponential fitting parameters of the PL decay traces of Eu-Ru(phen)$_3$-MOF and H$_3$L in the microsecond time window shown in the inset in Fig. 4b. (λ_{ex} = 465 nm)

	A_1	τ_1/ns	A_2	τ_2/ns
H$_3$**L**	474.694	1822		
Eu-Ru(phen)$_3$-MOF	553.088	108.7	269.850	548.3

Based on Supplementary Equation 2 and Supplementary Equation 3, the electron transfer time (t^2_{LMCT}) was 293.6 ns.

Supplementary Table 4. The bi-exponential fit parameters of the TA kinetics of H_3L and Eu-Ru(phen)$_3$-MOF in DMF shown in Fig. 4d

	A_1	τ_1/ns	A_2	τ_2/ns
H_3**L**	1.114×10^{-3}	0.2646	6.486×10^{-3}	54.27
Eu-Ru(phen)$_3$-MOF	4.989×10^{-4}	0.2341	4.237×10^{-4}	2.300

Based on Supplementary Equation 2 and Supplementary Equation 3, the electron transfer time (t^{3}_{LMCT}) was 1.2 ns

Supplementary Table 5. Single Crystal X-ray Structure Refinement of Eu-Ru(phen)$_3$-MOF

Empirical formula	$C_{120}H_{72}Eu_2N_{26}O_{22}Ru_2$
Formula weight	2736.09
Temperature/K	100(2)
Crystal system	orthorhombic
Space group	*I*222
a/Å	37.086(7)
b/Å	41.378(8)
c/Å	41.990(8)
α/°	90
β/°	90
γ/°	90
Volume/Å^3	64435(22)
Z	8
ρ_{calc}mg/mm^3	0.564
μ/mm^{-1}	0.506
F(000)	11072.0
Index ranges	$-27 \le h \le 33$, $-29 \le k \le 24$, $-37 \le l \le 29$
Reflections collected	33516
Independent reflections	19190 [R_{int} = 0.0501, R_{sigma} = 0.0679]
Data/restraints/parameters	19190/1192/1477
Goodness-of-fit on F^2	0.921
Final R indexes [I>=2σ (I)]	R_1 = 0.0451, wR_2 = 0.0958
Final R indexes [all data]	R_1 = 0.0694, wR_2 = 0.1014
Largest diff. peak/hole/e Å^{-3}	0.17/-0.20

$R_1 = \Sigma| |F_o|-|F_c| |/ \Sigma|F_o|$, $wR_2 = [\Sigma w(F_o^2-F_c^2)^2]/ \Sigma w(F_o^2)^2]^{1/2}$

Supplementary Note 1

Gas adsorption experiment with activation

The crystals of Eu-Ru(phen)$_3$-MOF were washed with DMF and MeOH several times, and then soaked in MeOH for 24 hours. After methanol exchange three times, the sample was heated under vacuum at 100 ^{o}C for 3 hours. The resulting sample was used to perform gas uptake measurements. Framework distortions were observed during the BET measurement, which is a common phenomenon for MOFs with large open channels.[1-2] The framework distortions can reasonably account for the low value of N_2 uptake and irregular adsorption/desorption curves.

Supplementary Note 2

Proposed TEOA degradation in photocatalytic reaction

In the photocatalytic reaction, TEOA was widely used as sacrificial agent which can provide electron and hydrogen proton.[3-5] A previous study also showed that the presence of a base in the reaction system supports the hydrogenation of CO_2 to formate.[6] Apparently, in addition to acting as an electron donor, TEOA can facilitate the photocatalytic CO_2 reduction because of its more basic nature. As a sacrificing agent, a small amount of TEOA should be degraded during the reaction. As shown in Supplementary Fig. 18b, one additional peak at m/z = 148.0905 was observed in the spectrum after reaction. Based on the previous report,[7] this additional peak associated to the product could be assigned to an aldehyde, which arises as a result of TEOA oxidation (Supplementary Fig. 19).

Supplementary Note 3

Electron transfer time calculations

The PL decays fitting by a bi-exponential function: $D(t) = A_1 \exp(-t/\tau_1) + A_2 \exp(-t/\tau_2)$, where $D(t)$ is the observed intensity at time t, A is amplitude and τ is time constant of the fast (index = l) and the slow (index = 2) components.

The averaged lifetime was calculated according to the Supplementary Equation 1,

$$t = \frac{A_1\tau_1 + A_2\tau_2}{A_1 + A_2} \qquad (1)$$

Fitting of the above data to Supplementary Equation 1, lifetimes 21.39 ns for H_3**L** (t_0) and 4.72 ns

for Eu-Ru(phen)$_3$-MOF (t_1) were obtained. Furthermore, based on the emission lifetimes of H$_3$**L** and Eu-Ru(phen)$_3$-MOF, we can obtain the rate of electron transfer to Eu$_2$ clusters k_{ET} via Supplementary Equation 2.[8]

$$k_{ET} = \frac{1}{t_1} - \frac{1}{t_0} \qquad (2)$$

Supplementary Note 4

Energy level calculations

LUMO energy level of ligand was calculated according to the equation[9]: $E^* = E - E'/q$, where E^* is the LUMO energy level and q (=1) is the charge transferred for Ru(III)/Ru(II) couple. E' is energy difference between HOMO and LUMO orbital, calculated from the luminescence emission peak at 598 nm of H$_3$**L** shown in Supplementary Fig. 24a. E is the redox potential level of Ru(III)/Ru(II) couple at the underground state. The value of E was determined from the cyclic voltamogram shown in Supplementary Fig. 24b.

To gain a deeper understanding of the electron transfer behavior, electrochemical experiments were performed. Mott-Schottky equation[10] was employed to calculate the built-in potential of the flat-band potential of Eu_2O_3 in the form of Supplementary Equation 3.

$$\frac{1}{C^2} = \frac{2}{\varepsilon_0 \varepsilon A^2 N_D}(V - V_{fb} - \frac{k_B T}{e}) \qquad (3)$$

Here C is the space charge capacity (F), A is the photoelectrode area (cm^2), ε_0 (= 8.854×10^{-12} F m^{-1}) is the permittivity of free space, ε is the relative permittivity of the Eu_2O_3, V is the applied voltage (V), V_{fb} is the flat band potential (V), N_D is the carrier concentration of Eu_2O_3, k_B (=8.6173324×10^{-5} eV K^{-1}) is the Boltzmann's constant, T is the temperature (K), and e is (=1.60×10^{-19} C) the elemental charge. From the plot of 1/C^2 *vs.* V, the N_D and V_{fb} can be obtained. Here, The Mott−Schottky measurements on Eu_2O_3 were conducted at frequencies of 0.5kHz, 1.0 kHz and 1.5kHz (Supplementary Fig. 25a) for investigating its semiconductor character. The positive slope of the obtained C^{-2} values (*vs.* the applied potentials) is consistent with that of the typical n-type semiconductors. The intersection point is independent of the frequency, and the flat band position (V_{fb}) determined from the intersection is -0.78 V. Since it is generally believed that the bottom of the conduction band in n-type semiconductors is more positive by about 0.10 V than the flat band potential,[11] the CB of Eu_2O_3 can be estimated to be -0.68 V *vs*. NHE, which is more negative than the reduction potential of CO_2/HCOOH (-0.58 V vs. NHE).[12]

Supplementary Methods

Synthesis of 1,10-Phenanthroline-5,6-dione

An ice cold mixture of concentrated H_2SO_4 (40 mL) and HNO_3 (20 mL) was added to 4.0 g of 1,10-phenanthroline and 4.0 g of KBr. The mixture was heated at reflux for 3 h. The hot yellow solution was poured over 500 mL of ice and neutralized carefully with NaOH until neutral to slightly acidic pH. Extraction with $CHCl_3$ followed by drying with Na_2SO_4 and removal of solvent gave 4.5 g (21.4 mmol, 96% yield) of 1,10-Phenanthroline-5,6-dione. The solid was purified further by crystallization from ethanol. ^{1}H NMR (500 MHz, DMSO): δ 9.10 – 8.94 (m, 2H), 8.40 (dd, J = 7.8, 1.5 Hz, 2H), 7.68 (dd, J = 7.8, 4.6 Hz, 2H)..

Synthesis of 4-(1*H*-imidazo[4,5-f][1,10]phenanthrolin-2-yl)benzoic acid (HNCP)

10-Phenanthroline-5,6-dione (0.1 mmol, 210 mg) was dissolved, together with 4-carboxybenzaldehyde (0.1 mmol, 150 mg), in 10 mL of acetic acid. The mixture system temperature was increased to 100 ℃ and the mixture stirred for 30 min, to which 1.46 g of dry ammonium acetate (19.0 mmol, 20 equiv) was added with stirring at 120 ℃ for 2 h. Then the reaction system was reduced to room temperature, filtered, and washed with a lot of water, acetone, and ether to give the desired 4-(1*H*-imidazo[4,5-f][1,10]phenanthrolin-2-yl)benzoic acid, (286 mg, 0.84 mmol, 88% yield). ^{1}H NMR (500 MHz, DMSO-d^6): δ 13.98 (s, 1H), 9.07 (s, 2H), 8.96 (d, J = 7.0 Hz, 2H), 8.42 (d, J = 8.5 Hz, 2H), 8.19 (d, J = 8.6 Hz, 2H), 7.87 (ddd, J = 25.1, 7.9, 4.2 Hz, 2H). ^{13}C NMR (126 MHz, DMSO-d^6): δ 167.40 (s), 149.80 (s), 148.20 (s), 143.92 (s), 134.10 (s), 131.72 (s), 130.38 (s), 130.20 (s), 126.57 (s), 123.67 (s).

Synthesis of metalloligand H_3L

130 mg $RuCl_3 \cdot 3H_2O$ (0.5 mmol), 511 mg HNCP (1.5 mmol) and 20 mL ethylene glycol were added into a 50 mL neck round bottom flask with stir bar and then heated at 180 ℃ for 10 hours under N_2. Then the mixture was cooled and poured into 50 ml water, followed by 20 mL of saturated KPF_6 in methanol was added drop wise under stirring and large quantity of oranges solids were obtained. After stirring 30 minutes, the precipitate was filtered and washed with water. The black solid (ca.650 mg) was obtained after drying under vacuum. The solid was dissolved in a mixture of 30 mL ethanol, 30 mL THF and 30 mL of 3 M NaOH (aq) and then refluxed at 80 ℃ overnight. The solution was then cooled to room temperature and acidified to pH = 1 with HCl.

The suspension was stored 1 hour and the precipitate was filtered, washed with water and then dried under vacuum to give pure H_3**L** (490mg, 0.437 mmol, 87% yield). ^{1}H NMR (500 MHz, DMSO-d^6): δ 9.01 (d, J = 8.5 Hz, 6H), 8.25 (d, J = 8.2 Hz, 6H), 7.89 (d, J = 8.3 Hz, 6H), 7.80 (d, J = 4.3 Hz, 6H), 7.63 – 7.50 (m, 6H). 13C NMR (126 MHz, DMSO-d^6): δ 171.79 (s), 161.86 (s), 147.08 (s), 144.67 (s), 138.90 (s), 138.13 (s), 137.43 (s), 130.07 (s), 129.74 (s), 126.32 (s), 126.07 (s), 125.16 (s). ESI-MS: cald for $C_{60}H_{36}N_{12}O_6Ru$ $[M-3H]^{-}$ 1119.17, found 1119.16, $[M-4H]^{2-}$ 559.07, found 559.08.

Synthesis of Pr-Ru(phen)$_3$-MOF

This compound was prepared using the same procedure as described above for the synthesis of its Eu(III) cognate but using $Pr(NO_3)_3$ $6H_2O$ in place of $Eu(NO_3)_3$•$6H_2O$. Yield: 3 mg (43%). FT-IR (3500−400 cm^{-1}): 3065 (br), 1660 (m), 1595 (s), 1380 (s), 1190 (w), 1092 (w), 1020 (w), 803 (m) 722 (m), 650 (w), 534 (m), 423 (w).

Synthesis of [$Eu_2(MMA)_6(H_2O)_4$]

The discrete Eu_2 cluster was hydrothermally prepared from a mixture of $Eu(NO_3)_3$ $6H_2O$ (0.2 mmol), methacrylic acid (0.6 mmol), NaOH (0.6 mmol) and H_2O (10 ml). The slurry was stirred for 30 min and heated at 393 K for 72 h in a Teflonlined stainless steel autoclave (25 mL) under autogenous pressure. After cooling to room temperature, the block-shaped crystals were washed with water and dried in air.

Surface photovoltage (SPV) measurements

Surface photovoltage spectroscopy measurements were conducted under vacuum (8.6 x 10^{-5} mbar) on films with area of 0.7 cm^2 deposited on indium−tin oxide (ITO) substrates. The films of the samples were prepared by drop-coating 5 mg mL^{-1} aqueous (650 μL water, 250 μL isopropanol and 100 μL of Nafion solution) dispersions of the photocatalysts on ITO slides, then dried in air. A gold Kelvin probe (Delta PHI Besocke) mounted inside a vacuum chamber served as the reference electrode. The samples were illuminated with monochromatic light from a 150 W Xe lamp, filtered through an Oriel Cornerstone 130 monochromator. The light power is limited to 0.1–2.5 mW cm^{-2} to prevent SPV artefacts from excitation of the Kelvin probe under higher intensity.

Cyclic voltammetry for H_3L

Cyclic voltammetry was performed using a Potentiostat/Galvanostat Model 263A in DMSO solutions containing 0.1 M (Bu_4N) PF_6 as the supporting electrolyte, at a scan rate of 100 mV s^{-1}.

Platinum and glassy graphite were used as the working and counter electrodes, respectively, and the potentials were measured against an Ag/AgCl reference electrode. Ferrocene was used as an internal reference.

Mott-Schottky measurements for Eu_2O_3

The Mott-Schottky measurements were carried out in a three-electrode, single-compartment quartz cell. The as-prepared sample deposited on indium tin oxide (ITO) glass was the working electrode. (The slurry was prepared as follows: 3 mg Eu_2O_3 was dispersed in 650 μL EtOH and 250 μL isopropanol and 100 μL of Nafion solution). The counter and reference electrodes were platinum gauze and Ag/AgCl, respectively. And the experiment was performed at room temperature in 0.5 M Na_2SO_4 electrolyte (100 mL, pH = 6.8) deoxygenated using an N_2 stream.

Transient absorption spectroscopy

The femtosecond transient absorption setup used for this study was based on a regenerative amplified Ti: sapphire laser system from Coherent (800 nm, 35 fs, 6 mJ/pulse, and 1 kHz repetition rate), nonlinear frequency mixing techniques and the ultrafast transient absorption spectrometer (Time-Tech Spectra, femtoTA100). Briefly, the 800 nm output pulse from the regenerative amplifier was split in two parts with a 50% beam splitter. The transmitted part was used to pump a TOPAS Optical Parametric Amplifier (OPA) which generates a wavelength-tunable laser pulse from 250 nm to 2.5 m. Here a 400 nm laser was used as pump beam. The reflected 800 nm beam was split again into two parts. One part with less than 10% was attenuated with a neutral density filter and focused into a 3 mm CaF_2 window to generate a white light continuum (WLC) from 340 nm to 800 nm used for probe beam. The CaF_2 crystal is mounted on a continuously moving stage to avoid thermal damage. The probe beam was focused with an Al parabolic reflector onto the sample. After the sample, the probe beam was collimated and then focused into a fiber-coupled spectrometer with CMOS sensors and detected at a frequency of 1 KHz. The intensity of the pump pulse used in the experiment was controlled by a variable neutral-density filter wheel and kept to be 14 J/cm^2. The delay between the pump and probe pulses was controlled by a motorized delay stage. The pump pulses were chopped by a synchronized chopper at 500 Hz and the absorbance change was calculated with two adjacent probe pulses (pump-blocked and pump-unblocked).The sample dispersed in DMF was held in a 2 mm quartz curvette and stirred constantly by a magnetic stirrer during the measurements.

DFT calculations

Spin polarized DFT calculations were performed in the Vienna ab initio Simulation Package (VASP).[13] Perdew Burke Ernzerh of (PBE)[14] functional with generalized gradient approximation (GGA) were used to treat exchange-correlation (XC) effects. Projector-augmented wave (PAW) method of Blöchl featuring the accuracy of augmented plane-wave methods as well as the efficiency of the pseudopotential approach,[15] is used to described the interactions between ion and electrons. 4f electrons of Eu, 2*s* and 2*p* electrons of C and O, and 1s electrons of H were explicitly treated as valence electrons. The electron wave function is expanded in plane waves up to a cutoff energy of 550 eV. The integration in Brillouin zone applied Γ centred 2×2×2 grid, being enough accurate in the calculation of total energy. In the self-consistent field calculation, the convergence of total energy was 1.0×10^{-5} eV. For the correction of self-interaction error in PBE functional, the DFT+U strategy with the Hubbard-U of 7.3 eV were used.[16] Moreover, the dispersion interaction of van der Waals force was corrected by DFT-D3 method of Grimme.[17] Geometrical optimization was completed until the resultant Hellmann–Feynman force per atom being less than 0.03 eV/Å. The initial structures are derived from single-crystal diffraction results. To reproduce photocatalytic process, the calculation proceeded under the constraint of -2 charge for catalyst. The adsorption energy were typically calculated according to the formula of $E_a = E_{(CO2\text{-}Catalyst)} - E_{(CO2)} - E(Catalyst)$, where $E_{(CO2\text{-}Catalyst)}$, $E_{(CO2)}$ and $E_{(Catalyst)}$ are energy of relaxed structure.

Supplementary References

1. Ghoufi, A. & Maurin, G. Hybrid Monte Carlo Simulations Combined with a Phase Mixture Model to Predict the Structural Transitions of a Porous Metal-Organic Framework Material upon Adsorption of Guest Molecules. *J. Phys. Chem. C.* **114**, 6496-6502 (2010).

2. Wang, C., DeKrafft, K. & Lin, W. Pt Nanoparticles@Photoactive Metal-Organic Frameworks: Efficient Hydrogen Evolution via Synergistic Photoexcitation and Electron Injection. *J. Am. Chem. Soc.* **134**, 7211-7214 (2012).

3. Chen, D., Xing, H., Wang, C. & Su, Z. Highly efficient visible-light-driven CO_2 reduction to formate by a new anthracene-based zirconium MOF via dual catalytic routes. *J. Mater. Chem. A*, **4**, 2657-2662 (2016).

4. Zhang, H. *et al*. Efficient Visible-Light-Driven Carbon Dioxide Reduction by a Single-Atom Implanted Metal-Organic Framework. *Angew. Chem., Int. Ed.* **128**, 14522-14526 (2016).

5. Wang, D. *et al*. Fe-Based MOFs for Photocatalytic CO_2 Reduction: Role of Coordination Unsaturated Sites and Dual Excitation Pathways. *ACS Catal.* **4**, 4254-4260 (2014).

6. Himeda, Y. Conversion of CO_2 into Formate by Homogeneously Catalyzed Hydrogenation in Water: Tuning Catalytic Activity and Water Solubility through the Acid-Base Equilibrium of the Ligand. *Eur. J. Inorg. Chem*. **25**, 3927-3941 (2007).

7. Probst, B., Rodenberg, A., Guttentag, M., Hamm, P. & Alberto, R. A Highly Stable Rhenium-Cobalt System for Photocatalytic H_2 Production: Unraveling the Performance-Limiting Steps. *Inorg. Chem.*, **2010**, *49*, 6453-6460.

8. Ajayakumar, G., Kobayashi, M., Masaoka, S. & Sakai, K., Light-induced charge separation and photocatalytic hydrogen evolution from water using RuIIPtII-based molecular devices: Effects of introducing additional donor and/or acceptor sites. *Dalton Trans.*, **40**, 3955-3966 (2011)

9. Huang, X., Shen, Q., Liu, J., Yang, N. & Zhao, G. A CO_2 adsorption-enhanced semiconductor/ metal-complex hybrid photoelectrocatalytic interface for efficient formate production. *Energy Environ. Sci.*, **9**, 3161-3171 (2016).

10. Gelderman, K., Lee, L. & Donne, S. W. Flat-Band Potential of a Semiconductor: Using the Mott-Schottky Equation. *J. Chem. Educ.* **84**, 685 (2007).

11. Long, J. *et al*. Amine-functionalized zirconium metal-organic framework as efficient

visible-light photocatalyst for aerobic organic transformations. *Chem. Commun.*, **48**, 11656-11658 (2012).

12. Chang, X., Wang, T. & Gong, J. CO_2 photo-reduction: insights into CO_2 activation and reaction on surfaces of photocatalysts. *Energy Environ. Sci.*, **9**, 2177-2196 (2016).

13. Kresse, G. & Furthmüller, J. Efficient iterative schemes for *ab initio* total-energy calculations using a plane-wave basis set. *Phys. Rev. B.* **54**, 11169 (1996).G.

14. Perdew, J. P., Burke, K. & Ernzerhof, M. Generalized Gradient Approximation Made Simple. *Phys. Rev. Lett.*, **77**, 3865-3868 (1996).

15. Blohl, P. E. Projector augmented-wave method. *Phys. Rev. B.* **50**, 17953-17979 (1994).

16. Barbagallo, M. *et al* Experimental and theoretical analysis of magnetic moment enhancement in oxygen-deficient EuO. *Phys. Rev. B*, **81**, 235216 (2010).

17. Grimme, S. Semiempirical GGA-type density functional constructed with a long-range dispersion correction. *J. Comp. Chem.*, **27**, 1787-1799 (2006).
